# Household mobility responses to weather extremes in Kyrgyzstan

**DOI:** 10.1038/s41467-026-75052-2

**Published:** 2026-06-29

**Authors:** Barchynai Kimsanova, Thomas Herzfeld, Atabek Umirbekov, Kathleen Hermans, Daniel Müller, Nodir Djanibekov

**Affiliations:** 1 Leibniz Institute of Agricultural Development in Transition Economies (IAMO), Halle (Saale), Germany; 2 Martin-Luther-Universität, Halle-Wittenberg, Germany; 3 Humboldt-Universität zu, Berlin, Germany; 4 Integrative Research Institute on Transformations of Human-Environment Systems (IRI THESys), Humboldt-Universität zu, Berlin, Germany

**Keywords:** Economics, Developing world, Governance, Interdisciplinary studies

## Abstract

Weather extremes increasingly shape human mobility, yet their effects differ across households and locations. We advance understanding of climate-related mobility by distinguishing among four outcomes: domestic mobility, international mobility, combined domestic-international mobility, and immobility, rather than treating mobility as a simple binary choice. Focusing on Kyrgyzstan, a climate-vulnerable mountainous country in Central Asia, we combine nationally representative household panel data from 2013–2022 with high-resolution climate data. Here, we show that weather extremes, both within districts and in neighboring districts, are more strongly associated with international mobility and immobility than with domestic and combined domestic-international mobility. Responses differ substantially across households’ socioeconomic position, with immobility more common among households with lower socioeconomic position and more diverse mobility patterns observed among households with higher socioeconomic position. Our findings suggest that mobility responses to weather extremes in Kyrgyzstan are shaped by both geographic context and household socioeconomic position.

## Abstract (web summary)

Extreme weather in Kyrgyzstan is associated with higher international migration and immobility, with responses differing across household well-being levels and neighboring climate conditions.

## Introduction

When extreme weather events occur, such as severe droughts^1–3^, large-scale floodings^4,5^, or prolonged winter extremes^6^, people often move to other places, reflecting mobility responses to climatic stress^7,8^. Yet in many climate-vulnerable settings, people experience repeated losses while remaining in place^9,10^. This contrast is consistent with evidence suggesting that those most exposed to weather shocks often have fewer financial, health-related, and social resources available to support mobility^11–13^.

Mobility under climatic stress is shaped by household resources rather than constituting an automatic response to extreme weather. Evidence suggests that whether mobility occurs is associated with households’ ability to finance relocation, manage risk, and cope with uncertainty^14^. When resources are limited, financial, social, and emotional barriers may increase the costs associated with mobility^15–17^. Consequently, mobility toward safer or more prosperous destinations may be more feasible for households with sufficient resources to bear the costs of relocation and adjustment^18^.

Household resources shape not only whether mobility occurs, but also the distance of mobility. Among households with greater socioeconomic resources, severe weather shocks are often associated with a shift from local relocation to longer-distance mobility^19–22^, whereas households with fewer resources are more likely to remain in place or engage in shorter-distance mobility. Extreme weather can deplete savings^23^, destroy productive assets^24^, and weaken social networks^25^, leaving many households exposed while remaining in place^26^. Consequently, weather shocks may widen inequalities in mobility by facilitating long-distance movement among households with greater resources while reinforcing immobility among households with fewer resources^3^.

At the same time, immobility cannot always be explained by economic constraints alone. Evidence from diverse settings suggests that place attachment, identity-based ties to land and livelihoods, and gendered social roles linked to subsistence activities may reinforce staying despite increasing climatic risks^27^. For some households, ancestral land, social obligations, and cultural continuity outweigh the perceived benefits of mobility, even under repeated weather extremes^28^. These socially embedded forms of immobility can coexist with economic constraints, shaping mobility outcomes in ways that are not fully captured by income or asset thresholds alone^7,27,29^.

Mobility constraints linked to limited household resources may be particularly pronounced in mountain regions, where geographic conditions can increase the costs and risks of mobility^30–32^. High-altitude areas often experience rapid climatic change, including erratic precipitation, harsher winters, and prolonged droughts that can place sustained pressure on farming and pastoral livelihoods^33,34^. At the same time, rugged terrain, long travel distances, sparse infrastructure, and limited market access may reduce nearby relocation options and increase the fixed costs of mobility. Consequently, weather extremes in mountain regions may constrain mobility rather than induce it, leaving households with fewer resources less able to move, while those with greater resources may be better positioned to mobilize the resources required for relocation^35^.

**Fig. 1:**
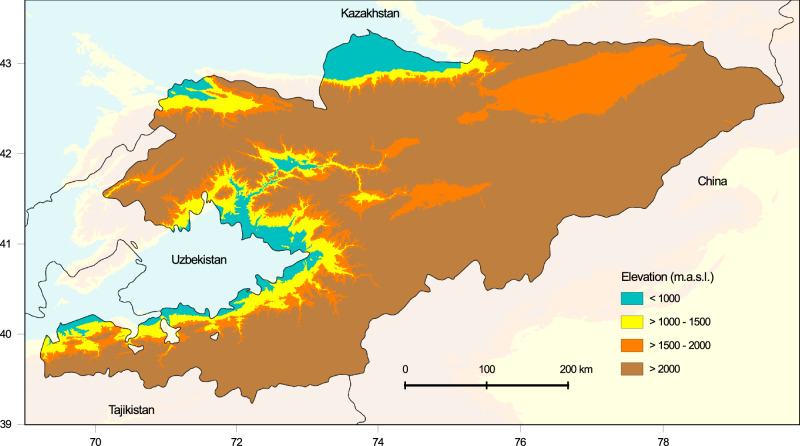
Elevation map of Kyrgyzstan. Elevation shown in four discrete elevation bands, measured in meters above sea level (m.a.s.l.).

Kyrgyzstan is highly exposed to climate change, with significant warming trends and changing precipitation and runoff patterns^36^. Climate projections indicate that the frequency and intensity of droughts and heavy rainfall events are likely to increase in the coming decades^37^. These trends are expected to place increasing pressure on water resources, agriculture, and natural hazard risks across the country’s predominantly mountainous terrain, potentially increasing the vulnerability of rural livelihoods^38^. Against this backdrop, Kyrgyzstan provides a useful context to examine how weather extremes interact with household mobility under high-altitude conditions (Fig. 1). Approximately 90% of the country’s territory lies above 1500 m, and nearly 10% of the population resides above 2000 m, where geographic isolation and exposure to weather extremes are particularly pronounced^39^. Labor migration plays a central role in livelihoods and risk management: in 2022, 16% of Kyrgyz citizens lived abroad and remittances accounted for nearly 20% of national GDP^40^. Nevertheless, geographic barriers, socioeconomic disadvantages, and limited accessibility may restrict migration opportunities for some highland communities^41^, while place attachment and strong social ties may also reinforce decisions to remain in place.

Constraints on mobility extend beyond income alone. Health, nutrition, education, and asset holdings also shape households’ ability to respond to weather extremes^42–44^. In mountain regions, remoteness and recurrent weather extremes can compound these disadvantages, contributing to precarious mobility or immobility^45^. Collectively, these dimensions reflect broader differences in household well-being, understood here as the living conditions and resources that shape households’ capacity to respond to climatic stress.

**Fig. 2:**
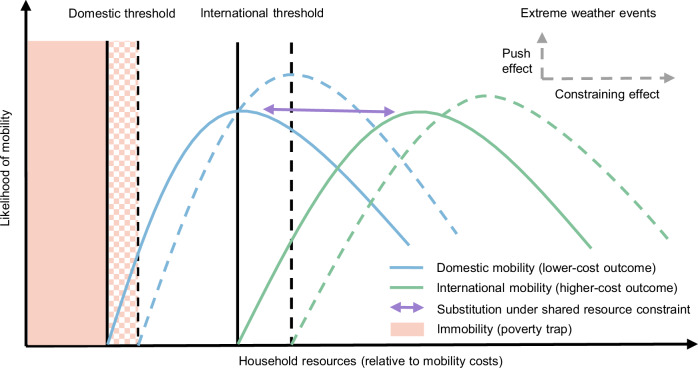
Conceptual illustration of resource-based mobility constraints under extreme weather. The figure presents a stylized conceptual framework rather than empirical results. Solid curves represent baseline mobility outcomes as household capabilities increase relative to mobility costs. Domestic mobility (blue curve) becomes feasible after a lower capability threshold, whereas international mobility (green curve) requires substantially greater capabilities. The purple arrow indicates substitution across mobility outcomes under a shared household constraint, whereby households allocate available resources toward higher-cost international mobility as capabilities increase. Dashed curves and dashed vertical lines illustrate how extreme weather events may shift mobility outcomes and thresholds by increasing incentives for mobility (push effects) or by tightening constraints through resource depletion or rising mobility costs (constraining effects). The shaded area denotes the range in which households remain immobile because available capabilities are insufficient to enable mobility.

Here, we examine how extreme weather, geography, and household well-being jointly shape household mobility and immobility in Kyrgyzstan. Household well-being is measured using a composite index encompassing nutrition, healthcare access, education, and living standards, developed by the authors and designed to capture broader dimensions of well-being beyond conventional income-based measures^46^. Our framework builds on the New Economics of Labour Migration^47^ and spatial poverty trap models^48^ and is related to aspirations-capability approaches to migration and climate adaptation^49^. Figure 2 conceptually illustrates this resource-threshold framework, in which domestic mobility becomes feasible at lower resource levels, whereas international mobility requires substantially greater capabilities, with households remaining immobile when neither threshold is reached (Supplementary Note 1 provides a full derivation). Weather extremes may shift these mobility outcomes by increasing incentives for mobility or by tightening resource constraints through asset loss and rising mobility costs. Using nationally representative longitudinal household data combined with high-resolution climate information, we distinguish among domestic mobility, international mobility, combined mobility (households experiencing both domestic and international mobility), and immobility. Our analysis highlights substantial heterogeneity in mobility outcomes across household well-being levels, geographic settings, and spatially interconnected climate exposures.

## Results

### Spatial and temporal variation in mobility, well-being, and weather extremes

**Fig. 3:**
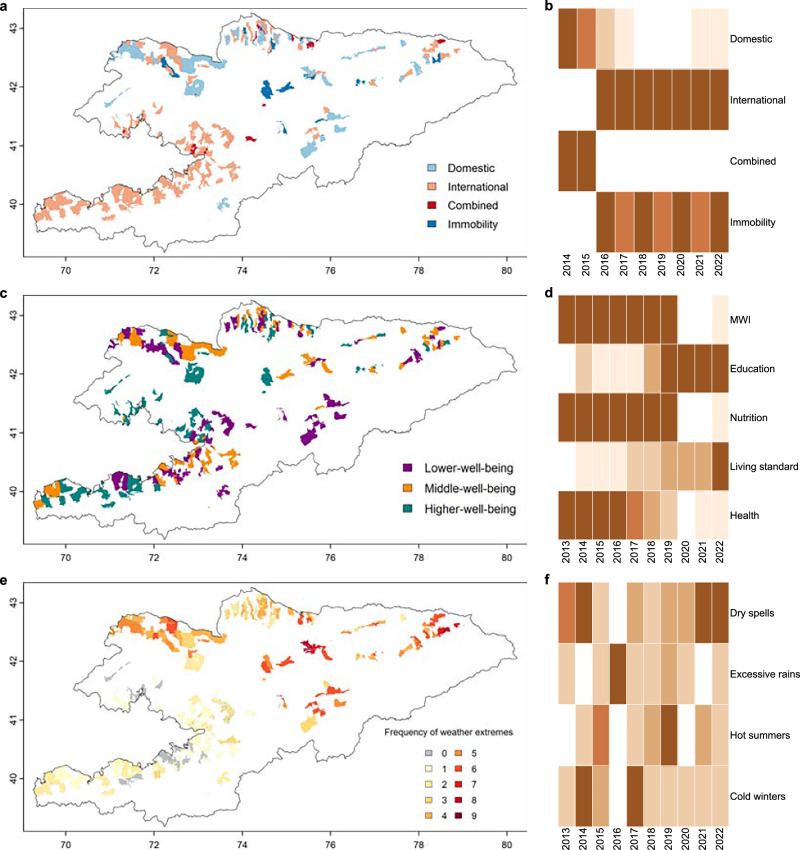
Spatial and temporal (2013–2022) variation in mobility, household well-being, and weather extremes in Kyrgyzstan. **a** Shows the spatial distribution of household mobility outcomes across districts, and **b** shows their temporal evolution over the study period. **c** Maps average household well-being across districts, while **d** presents temporal variation in average MWI and its component dimensions. **e**, **f** Illustrate the spatial frequency and temporal variation of weather extremes, including dry spells, excessive rains, hot summers, and cold winters. Spatial frequencies are measured as the number of years in which districts experienced at least one extreme event. Lighter shades indicate lower frequencies and darker shades indicate higher frequencies.

Mobility outcomes vary substantially across space and time (Fig. 3a, b). Domestic, international, combined mobility, and immobility are observed across Kyrgyzstan, with international mobility concentrated particularly in southwestern and several mid-elevation districts, while domestic and combined mobility occur more sporadically across the country (Fig. 3a). Temporal patterns indicate a decline in domestic and combined mobility after 2016, whereas international mobility became relatively more prominent in later years (Fig. 3b), coinciding with Kyrgyzstan’s accession to the Eurasian Economic Union, which expanded access to foreign labor markets^50^. Descriptive statistics further show that immobility is the most common outcome across all elevation groups, accounting for 78–93% of households depending on elevation, with the highest immobility rates observed among households located above 2000 m (Table 1).

**Table 1:** Descriptive statistics by elevation band

	<1000 m	1000–1500 m	1500–2000m	>2000m
*Mobility outcomes*				
Immobile (%)	89	78	87	93
Domestic (%)	3	4	4	2
International (%)	6	14	8	5
Combined (%)	2	4	2	1
*Household well-being*				
MWI (mean)	0.66	0.65	0.66	0.63
Lower-well-being (%)	32	40	34	51
Middle-well-being (%)	39	37	36	36
Higher-well-being (%)	29	23	30	14
*Extreme weather events*				
Dry spells-exposure (%)	11	4	14	11
Dry spells-intensity (mean)	1.24	1.21	1.41	1.39
Excessive rains-exposure (%)	36	30	33	26
Excessive rains-intensity (mean)	1.47	1.61	1.63	1.79
Hot summers-exposure (%)	53	51	56	46
Hot summers-intensity (mean)	1.61	1.54	1.73	1.83
Cold winters-exposure (%)	10	10	11	11
Cold winters-intensity (mean)	1.72	1.68	1.62	1.63
*Household characteristics*				
Household size (mean)	5	5	5	5
Dependency ratio (mean)	0.36	0.40	0.40	0.40
Male share (mean)	0.47	0.47	0.50	0.49
Households	2857	1025	1750	663
Observations (N)	17,436	7004	11,366	4344

The table summarizes household mobility outcomes, multidimensional well-being (MWI), household characteristics, and exposure to extreme weather across four elevation categories. Elevation bands are defined as <1000 m, 1000 ≤ x ≤ 1500 m, 1500 <x ≤ 2000 m, and >2000 m. Mobility outcomes are expressed as shares of households. Weather exposure variables report the share of households experiencing at least one extreme event, while intensity measures capture the average magnitude of climatic deviation (SPI/STI) conditional on exposure, averaged across households within each elevation band. All statistics are survey-weighted; reported observations (N) refer to unweighted sample size.

Household well-being also varies across space and time (Fig. 3c, d). For descriptive and analytical purposes, households are classified into lower-, middle-, and higher-well-being groups based on terciles of the multidimensional well-being index (MWI). Lower-well-being households are disproportionately concentrated in remote mountain districts (Fig. 3c). Because the index is constructed using a fixed set of indicators and weights, temporal variation reflects changes in underlying living conditions rather than changes in measurement. Consistent with this structure, average household well-being increased modestly in the early years of the panel but declined after 2020, reflecting deteriorations in nutrition and health during the COVID-19 period (Fig. 3d). Descriptive statistics further show that households located above 2000 m exhibit the lowest average well-being and the highest share of lower-well-being households (Table. 1), indicating a concentration of vulnerability in high-altitude areas.

Weather extremes are widespread and recurrent across Kyrgyzstan (Fig. 3e, f). Districts experience repeated dry spells, excessive rains, hot summers, and cold winters, with several northern and mountainous regions exposed to multiple extremes within the same year (Fig. 3e). Temporal variation further indicates substantial year-to-year fluctuations in the intensity of weather extremes across the study period (Fig. 3f). Exposure appears particularly persistent in high-elevation areas, which also exhibit lower levels of household well-being. These patterns suggest a spatial overlap between weather extremes and lower household well-being. International mobility is most prevalent among households located at mid-elevations (1000–1500 m), whereas combined mobility remains rare across all elevation categories (Table 1)

### Extreme weather intensity and mobility outcomes

**Fig. 4:**
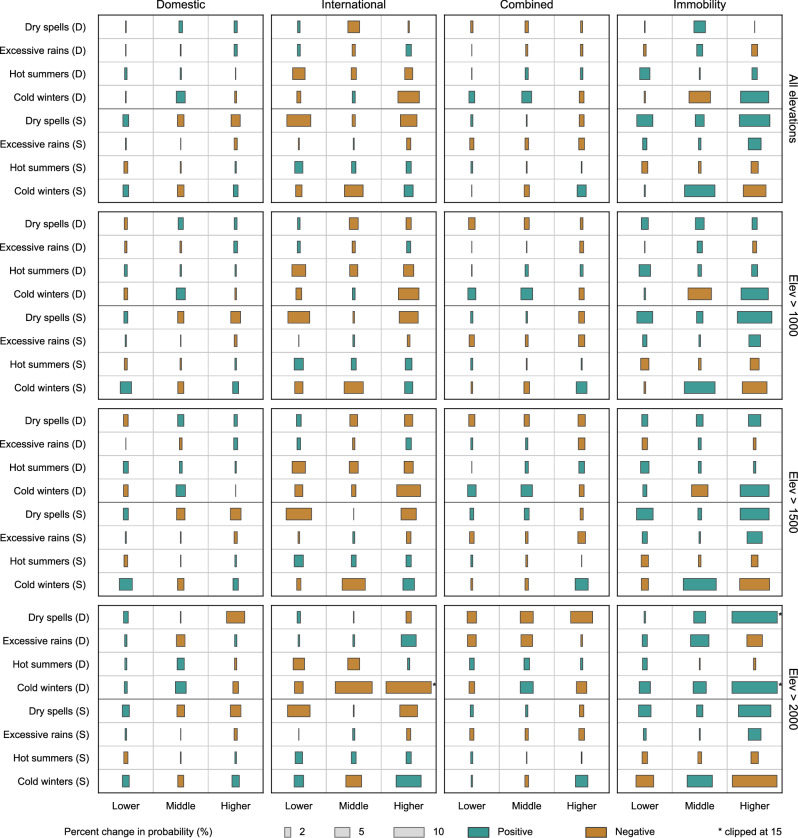
Associations between extreme weather intensity and household mobility outcomes. Average marginal associations between extreme weather intensity conditional on exposure and percentage-point changes in the predicted probabilities of household mobility outcomes (domestic mobility, international mobility, combined mobility, and immobility). Estimates are conditional on household well-being (MWI), household characteristics, and spatial and temporal controls, and incorporate all interactions included in the empirical specification. Weather variables are shown separately for direct exposure (D), measured in the household’s own district, and spillover exposure (S), measured as average intensity in neighboring districts. Results are presented across nested elevation bands (>1000 m, >1500 m, and >2000 m a.s.l.) and lower-, middle-, and higher-well-being groups, defined by terciles of the MWI distribution. Shading indicates the direction and magnitude of associations. Estimates reflect conditional associations and should not be interpreted as causal effects.

Associations between extreme weather intensity and household mobility outcomes are estimated for both direct exposure and spillover exposure from neighboring districts. Across nested elevation bands (i.e., progressively higher elevation thresholds where higher bands are subsets of lower ones) and household well-being groups, these associations vary in both magnitude and direction (Fig. 4). Associations are generally larger in magnitude for international mobility and immobility than for domestic and combined mobility. Full estimation results, average marginal effects, supplementary source tables, and the custom analysis code are provided in the Zenodo and Code Ocean repositories referenced in the Data availability and Code availability statements.

Associations between extreme weather intensity and domestic mobility are generally modest but vary across household well-being groups and exposure types. Conditional on exposure, more severe cold winters are associated with higher domestic mobility among middle-well-being households under direct exposure, with increases of about 3% across elevation bands. Similarly, greater spillover intensity of cold winters in neighboring districts is positively associated with domestic mobility among lower-well-being households, with increases of roughly 2–4%. In contrast, greater spillover intensity of dry spells is associated with lower domestic mobility among higher-well-being households (around −3%).

Associations between extreme weather intensity and international mobility are generally negative across household well-being groups and are largest in magnitude under spillover exposure. Among lower-well-being households, greater hot summer intensity is associated with lower probabilities of international mobility (approximately −3%), while greater spillover intensity of dry spells is associated with larger declines (−5% to −6%). Among higher-well-being households, greater cold-winter intensity under direct exposure is associated with reductions in international mobility (−7% to −12%), while middle-well-being households show similar declines under spillover cold-winter intensity (−5% to −7%). Overall, exposure to extreme weather across multiple districts is associated with lower probabilities of international mobility.

Associations between extreme weather intensity and combined mobility are generally small. Across most weather variables, estimated associations conditional on exposure are close to zero across household well-being groups. Two exceptions are observed for cold winters: greater cold-winter intensity under direct exposure is associated with a modest increase in combined mobility among middle-well-being households (about 3%), while greater spillover intensity of cold winters is positively associated with combined mobility among higher-well-being households (around 2%). These associations are slightly larger above 2000 m but remain small relative to other mobility outcomes.

Associations between extreme weather intensity and immobility are strongest at high elevations and are positive in most specifications. Above 2000 m, more severe cold winters are associated with higher immobility among higher-well-being households, corresponding to increases of about 9–16% in the predicted probability of immobility. Greater spillover intensity of cold winters in neighboring districts is also positively associated with immobility among middle-well-being households (around 8–11%). In addition, greater spillover intensity of dry spells is associated with higher immobility among higher-well-being households (around 11%), suggesting that spatially widespread climatic stress may be associated with a greater likelihood of remaining in place.

### Well-being and non-linear mobility outcomes

**Fig. 5:**
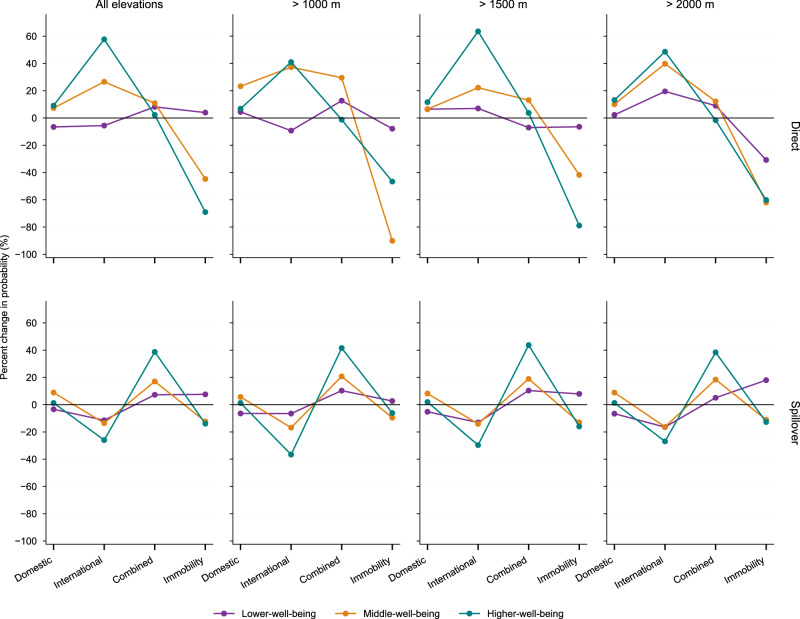
Multidimensional well-being and non-linear mobility outcomes. Average marginal associations between multidimensional well-being (MWI) and percentage-point changes in the predicted probabilities of immobility, domestic mobility, international mobility, and combined mobility, conditional on household characteristics and spatial and temporal controls. Estimates are shown separately for lower-, middle-, and higher-well-being groups, defined by terciles of the MWI distribution, illustrating non-linear patterns across the well-being distribution. Results also include spillover associations of MWI from neighboring districts. Estimates represent conditional associations and should not be interpreted as causal effects.

Figure 5 shows that household mobility outcomes vary across household well-being levels and display non-linear patterns. Immobility is most common among lower-well-being households, while domestic and combined mobility are most common among middle-well-being households. International mobility is most common among higher-well-being households. Spillover associations of household well-being from neighboring districts vary across mobility outcomes but change little across elevation bands. This pattern suggests that local household well-being is more closely associated with whether mobility occurs, whereas surrounding conditions may influence the type of mobility observed.

Associations between well-being and domestic mobility vary across household well-being groups, elevation, and spatial exposure. Among higher-well-being households, higher MWI is positively associated with domestic mobility under direct exposure, with effects ranging from about 6% to 13% across elevation bands. Spillover associations are smaller but remain positive across all elevations. Among middle-well-being households, spillover associations are positive across all elevations (6–9%), while direct associations are positive at higher elevations and become negative at lower elevations. In contrast, among lower-well-being households, higher MWI is associated with lower domestic mobility under both direct and spillover exposure, with larger negative associations at higher elevations, reaching roughly −14% (direct) and −7% (spillover).

For international mobility, associations with well-being vary across household well-being groups and spatial exposure. Among higher-well-being households, higher MWI is positively associated with international mobility under direct exposure, with effects ranging from about 50% to 80% and larger positive associations at higher elevations. Among middle-well-being households, direct associations are positive but smaller (15–30%), while among lower-well-being households they are negative across all elevations and larger in magnitude at higher elevations. Spillover associations are negative across all well-being groups and larger in magnitude than direct effects, particularly for higher-well-being households (around −25% to −35%). This pattern suggests that higher levels of household well-being in neighboring districts are associated with lower probabilities of international mobility.

For combined mobility, associations with well-being are positive across all household well-being groups but smaller in magnitude than those observed for international mobility and vary little across elevation bands. Spillover associations of well-being are largest in magnitude among higher-well-being households (38–44%), while middle-well-being households show positive spillover associations (17–21%). Lower-well-being households exhibit smaller positive spillover associations (7–10%). Direct associations between well-being and combined mobility are positive for all groups but generally smaller in magnitude, particularly among higher-well-being households, where effects remain below 7% and approach zero at some elevations.

Associations between well-being and immobility vary strongly across well-being groups and are largest in magnitude at higher elevations. Among lower-well-being households, higher MWI is positively associated with immobility under both direct and spillover exposure (4–17%). In contrast, middle- and higher-well-being households exhibit negative associations between MWI and immobility. Direct associations are particularly large among higher-well-being households, with the largest declines observed above 1500 m, where immobility decreases by approximately 57% to 98%. Spillover associations are also negative but smaller in magnitude. Overall, higher-well-being is associated with lower probabilities of immobility among middle- and higher-well-being households, whereas positive associations are observed among lower-well-being households. This pattern suggests non-linearities in the relationship between well-being and mobility outcomes.

## Discussion

Research on mobility in mountainous regions provides crucial insights into adaptation, livelihoods, and place attachment, yet nationally representative quantitative evidence remains scarce^45^. We examine how extreme weather, geography, and household capabilities are associated with mobility and immobility across a high-altitude country using nationally representative longitudinal household data. Our findings suggest that exposure to extreme weather alone is insufficient to explain mobility outcomes and that mobility responses are associated with differences in household well-being.

Our framework suggests that differences in access to material resources, healthcare access, education, and living standards are associated with different mobility outcomes under climate stress. We observe non-linear patterns in the relationship between household well-being and mobility outcomes, consistent with theoretical perspectives that view mobility as contingent on both aspirations and capabilities.

A key implication of our findings is that immobility cannot be interpreted solely as a failure to adapt or as a direct outcome of deprivation. Instead, immobility may represent a distinct outcome associated with both structural constraints and place-based preferences^51^. This pattern is consistent with evidence on voluntary immobility, whereby households remain in place due to place attachment, cultural identity, social obligations, and perceived risks associated with mobility. In mountain regions, attachment to land, pastoral livelihoods, and community cohesion may outweigh the perceived benefits of mobility, even under increasing climatic stress^7,27,29^.

At the same time, mobility decisions are associated with factors beyond material resources, including social networks, cultural attachment, and migration regimes that influence access to mobility opportunities. Our framework does not claim that multidimensional well-being fully explains mobility behavior. Rather, it provides a parsimonious and policy-relevant proxy for the capacity to mobilize resources required for relocation. Future research could integrate cultural, social, and institutional dimensions with quantitative measures of household resources to provide a more comprehensive understanding of mobility and immobility under climatic stress.

Our findings also carry important policy implications. First, climate adaptation and disaster risk management strategies should explicitly account for both mobility and safe immobility. Constrained or involuntary immobility may require targeted support rather than being treated as a residual outcome or policy failure^52^. Second, simultaneous weather extremes across neighboring districts are associated with lower probabilities of international mobility, suggesting the potential value of scalable in-situ support, including social assistance, healthcare access, and emergency services, especially in remote and high-elevation areas. In the Kyrgyz context, where international mobility – primarily to Russia and Kazakhstan – constitutes an important livelihood strategy, these findings suggest that mobility channels may become less accessible during periods of spatially widespread climatic stress, highlighting the importance of complementary domestic adaptation measures.

A third policy implication concerns the association between household well-being and mobility outcomes. Improvements in healthcare access, education, and living standards may expand the range of adaptation options available to households, including both voluntary mobility and safe immobility. This perspective aligns with National Adaptation Plan processes and Green Climate Fund-supported programming, which increasingly emphasize social protection, human development, and deprivation reduction as components of climate adaptation, particularly for populations facing structural mobility constraints^53^.

Finally, the importance of spatial spillovers suggests that adaptation planning may benefit from extending beyond administrative boundaries. Coordinated responses across climatically connected districts may be more effective than isolated interventions. Overall, our findings suggest that climate-related mobility cannot be understood through climatic exposure alone. Policies that jointly consider climatic stress, spatial interdependence, and household capabilities may be better positioned to support equitable and effective adaptation under increasing climate risk.

## Methods

### Scope and data coverage

We examine how socioeconomic conditions and extreme weather events are associated with household mobility outcomes in Kyrgyzstan during 2013–2022. The analysis draws on the Kyrgyz Integrated Household Survey (KIHS), a nationally representative quarterly rotating panel covering approximately 5000 households per year across all seven provinces and the capital city, Bishkek. Although the KIHS sampling design does not oversample high-altitude communities, it provides nationwide coverage that includes mountainous and remote areas. The study uses anonymized secondary survey data collected by the National Statistical Committee of the Kyrgyz Republic and therefore did not require additional institutional ethics approval or informed consent procedures by the authors.

The survey contains detailed information on household mobility, demographics, education, health, employment, expenditures, and asset ownership, with explicit stratification by urban and rural residence. District-level administrative identifiers (aiyl aymak) are available for all household–year observations used in the analysis, enabling the merging of household data with district-level spatial covariates for all 205 administrative districts. The estimation sample includes approximately 97% of surveyed households; observations lacking geographic identifiers are excluded.

KIHS follows a rotating panel design, and households are therefore not observed continuously over the full study period. To document the resulting panel structure, Supplementary Table 1 reports pairwise household retention rates across survey waves. Retention is high between adjacent years and declines gradually over longer intervals, reflecting the survey’s rotation design rather than selective attrition. Despite this rotation, the estimation sample is dominated by repeatedly observed households: the median household appears in eight survey waves, the mean participation length exceeds six years, and fewer than 1% of households exhibit non-consecutive observation gaps.

To measure weather extremes, we combine household data with high-resolution climate information from the ERA5-Land reanalysis product^54^, which provides monthly precipitation and temperature estimates at approximately 9 km spatial resolution. Climate variables are aggregated to the district level and aligned with the Central Asian hydrological year. We focus on four weather extremes that are particularly relevant in Kyrgyzstan: dry spells, excessive rains, cold winters, and hot summers.

Evidence from the Life in Kyrgyzstan (LiK) survey (2010–2019) indicates that dry spells, excessive rains, and cold winters are the most frequently reported climate-related shocks experienced by households, with 33% reporting excessive rains, 25% cold winters, and 16% dry spells during the observation period^55^. We also include hot summers, which have become more frequent under regional warming trends^56^, thereby complementing self-reported experiences with standardized climate metrics. For each hazard, we distinguish between exposure (the occurrence of an extreme event) and intensity (the magnitude of climatic deviation conditional on exposure), allowing us to assess whether household mobility is associated primarily with the occurrence of extreme conditions or with their severity once they occur.

### Key variables

We define household mobility as a four-category outcome: immobility (no residential change), domestic mobility (moves within Kyrgyzstan), international mobility (moves abroad), and combined mobility (moves within Kyrgyzstan and abroad). The KIHS records annually whether any household member changed residence during the preceding 12 months and whether the move occurred domestically or internationally. A household is classified as mobile if at least one member reports a residential change, with the mobility outcome determined by the reported destination(s). Some households report both domestic and international mobility by different members. Rather than excluding these cases or imposing a hierarchy across destinations, we classify such households as exhibiting combined mobility. This category captures within-household diversification of mobility and avoids conflating distinct mobility responses.

We measure household well-being using an MWI adapted from the Multidimensional Poverty Index framework of Alkire and Santos^57^ and previously developed for Kyrgyzstan^46^. The index is continuous, ranges from 0 to 1, and aggregates ten indicators across four equally weighted dimensions: nutrition, education, health, and living standards. Several adaptations reflect the Kyrgyz context. We exclude child mortality, included in the original Alkire-Santos index, due to its low incidence in Kyrgyzstan. We measure nutrition using dietary diversity based on a Berry Index constructed from food expenditure shares. The education dimension reflects compulsory schooling through preschool attendance and completion of at least eleven years of education by the household head. Health is captured through illness incidence, access to medical services, and the ability to cover healthcare costs, reflecting the importance of out-of-pocket expenditures in Kyrgyzstan. Living standards are measured using income and living space for all households, supplemented by land and livestock ownership for rural households, which better differentiate welfare in a post-Soviet setting. Supplementary Table 2 provide full details on index construction, indicators, and weighting. Collectively, these dimensions capture household living conditions relevant to mobility capabilities.

In this study, “well-being” is operationalized in a bounded, multidimensional sense as household living conditions relevant to mobility. The MWI captures material and service-related dimensions—nutrition, education, health, and living standards—rather than subjective, psychological, or broader relational aspects of well-being. We therefore interpret the index as a proxy for capability-related conditions associated with feasibility of mobility, rather than as a comprehensive measure of welfare.

We measure exposure to extreme weather events using the Standardized Precipitation Index (SPI) and the Standardized Temperature Index (STI), which capture monthly deviations from long-run historical climate averages on a standardized scale^58^. We focus on four climate stressors relevant for Kyrgyzstan, distinguished by their seasonal timing and underlying climatic mechanisms. We identify dry spells using SPI values below −1 calculated over the hydrological year (October–September), capturing sustained moisture deficits. We measure excessive rains using spring SPI values above 1, and we define cold winters and hot summers using January–February STI values below −1 and July–August STI values above 1, respectively.

We model exposure using continuous SPI and STI values and set values within the normal range (−1 to 1) to zero, such that only deviations beyond the onset of extreme conditions enter the analysis. This specification isolates the intensity of weather extremes conditional on exposure, allowing us to distinguish severity of extreme conditions from their occurrence. We aggregate dry-spell SPI at the provincial level because droughts operate over broad hydrological basins and district-level precipitation anomalies may not fully capture downstream water availability^59^. In contrast, excessive rains, cold winters, and hot summers are measured at the district level, where localized climatic deviations directly affect households. Supplementary Table 3 reports full variable definitions, measurement, and data sources.

### Econometric strategy

Household mobility outcomes are associated not only with household characteristics and local conditions, but also by conditions in surrounding areas that share labor markets, infrastructure, migration networks, and exposure to climatic shocks. These interdependencies can generate spatial clustering in mobility behavior, such that mobility outcomes in one district may be associated with conditions in neighboring districts. To capture this spatial dependence in a multi-category setting, we estimate an augmented multinomial logit (MNL) model. This approach extends the standard multinomial logit framework by allowing mobility probabilities to depend on both local covariates and spatially lagged explanatory variables.

Each household-year observation is classified into one of four mutually exclusive mobility outcomes: domestic mobility ($j=1$), international mobility ($j=2$), combined domestic and international mobility ($j=3$), and immobility ($j=4$), which serves as the reference category. The model estimates associations between household well-being, extreme weather intensity conditional on exposure, elevation, household characteristics, spatial spillovers, and the probability of each mobility outcome relative to immobility.

We model spatial dependence through spatially lagged covariates rather than spatially lagged dependent variables, allowing household mobility outcomes to be associated with surrounding conditions without imposing behavioral diffusion in outcomes themselves. We distinguish between socioeconomic spillovers and climatic spillovers, reflecting the different mechanisms through which these processes may operate.

For household socioeconomic characteristics, including MWI, we construct spatial lags using a K-nearest neighbors (KNN) travel-cost connectivity matrix. Each district is linked to its five nearest neighbors ($K=5$) based on effective travel costs rather than straight-line distance. Travel costs are derived from a global friction surface that integrates road networks, land cover, slope, and transport infrastructure to estimate least-cost travel times across terrain^60^. Pairwise weights are defined as the inverse of travel cost and row-standardized so that weights sum to one for each district. This approach is intended to represent effective socioeconomic connectedness, which is particularly important in mountainous settings where Euclidean distance may poorly reflect accessibility, market integration, and migration networks.

For climate variables, we construct spatial lags using a separate K-nearest neighbors matrix based on geographic proximity. Each district is linked to its five closest neighboring districts using inverse-distance weights computed from great-circle (haversine) distances. These weights are also row-standardized. This specification is intended to represent the spatial correlation and propagation of meteorological processes across neighboring districts, independent of transport infrastructure or economic connectivity. Spatial associations may reflect not only indirect spillover processes, but also shared regional exposure to correlated climatic conditions across neighboring districts. All spatially lagged variables are standardized to ensure comparability with their corresponding direct effects.

To capture geographic heterogeneity in mountain environments, we incorporate nested elevation bands (above 1000 m, 1500 m, and 2000 m a.s.l.) derived from a digital elevation model. The 2000 m threshold reflects a commonly used cutoff in mountain research, above which climatic conditions, accessibility constraints, and livelihood systems change sharply in Kyrgyzstan^61^. We interact elevation indicators with both extreme weather intensity and household well-being, allowing climate–mobility and well-being–mobility associations to vary across elevational gradients.

Household-level controls include household size, dependency ratio, gender composition (male share), and rural residence. We include year indicators to absorb common temporal shocks and macroeconomic trends. MWI is lagged by one period so that mobility outcomes are associated with pre-existing well-being conditions rather than contemporaneous changes driven by mobility itself. This lag structure reduces simultaneity concerns, although it does not fully eliminate possible feedback mechanisms through which migration may subsequently affect household welfare, for example via remittances or labor reallocation. We do not include district fixed effects, as these would absorb the spatial variation required to identify spillover effects; instead, spatial lags and elevation interactions capture geographic heterogeneity directly. All models are estimated using KIHS sampling weights.

To allow for non-linear relationships between well-being and mobility, we estimate the model separately for households in the lower-, middle-, and higher-well-being groups, defined by terciles of the MWI distribution, thereby avoiding the imposition of homogeneous effects across the well-being distribution. Uncertainty is assessed using a cluster bootstrap at the survey primary sampling unit (PSU) level, resampling PSUs with replacement and re-estimating the model in each replication. Bootstrap standard errors are computed as the standard deviation of coefficient estimates across replications, accounting for within-PSU dependence induced by the survey design.

A simplified representation of the estimated model is:

1 $${{\mathrm{ln}}}\left(\frac{P\left(Y=j\right)}{P\left(Y=4\right)}\right)=	 f\left({{{{\rm{MWI}}}}}_{t-1},\,{{{\rm{weather\; intensity|exposure}}}},{{{\rm{elevation}}}},\right.\\ 	\left.{{{\rm{spatial\; spillovers}}}},{{{\rm{controls}}}}\right),{j}=1,\,2,\,3.$$

The full mathematical specification is provided in Supplementary Methods 1.

### Robustness and sensitivity

We conduct a comprehensive set of robustness and sensitivity analyses to assess the stability of our findings to alternative specifications, measurement choices, spatial assumptions, and potential sources of bias. These checks address concerns related to simultaneity between household well-being and mobility, omitted variables, the definition and aggregation of weather extremes, spatial dependence, and sample composition in a rotating panel. Unless otherwise noted, all robustness exercises yield qualitatively similar patterns to the baseline results.

A key concern in studies linking household well-being to mobility outcomes is potential simultaneity, as mobility may itself affect well-being through remittances, labor loss, or selection into mobility. Our baseline specification mitigates this concern by lagging household well-being by one year ensuring that mobility outcomes are associated with pre-existing household conditions rather than contemporaneous changes driven by mobility. Nevertheless, migration may still subsequently affect household welfare through remittances, labor reallocation, or changes in household composition.

To further assess potential reverse causality, we estimate auxiliary regressions in which current household well-being is regressed on future mobility indicators. Results show that future mobility is associated with a small but statistically significant decline in current well-being, even after controlling for household demographics, elevation, and year effects (Supplementary Table 4). This pattern suggests that mobility is more likely to be preceded by lower well-being rather than driving contemporaneous improvements, supporting our lag structure. While these checks cannot fully eliminate endogeneity concerns, they indicate that reverse causality is unlikely to be the primary driver of the estimated associations. We therefore interpret all results as conditional associations rather than causal effects.

To assess sensitivity to the construction of household well-being, we re-estimate all models using an alternative index that excludes asset-based components and focuses on consumption, health, and education. This addresses concerns that asset ownership may mechanically correlate with long-term location choices or mobility capacity. Results remain highly stable: coefficient signs remain unchanged in more than 94% of cases across mobility outcomes, elevation bands, and MWI tercile groups, and confidence intervals overlap throughout. Although some magnitudes attenuate modestly, the non-linear patterns linking well-being to mobility, and immobility remain intact.

Household mobility outcomes in Kyrgyzstan exhibit strong spatial clustering, reflecting shared climatic conditions, infrastructure constraints, and migration networks. We formally assess spatial dependence using Moran’s I statistics computed on district-level averages of both mobility outcomes and weather variables. Results indicate strong and statistically significant spatial autocorrelation across all outcomes and climate variables, with Moran’s I values ranging between 0.51 and 0.91 across alternative neighborhood sizes (*K* = 4–10; Supplementary Table 5). These findings confirm that ignoring spatial structure would result in misspecified models.

To account for spatial dependence in mobility outcomes and climate exposure, our spatial multinomial logit framework incorporates spatial spillovers through spatially lagged covariates rather than spatially lagged dependent variables. Socioeconomic spillovers are modeled using a travel-cost-based KNN matrix derived from a global friction surface, capturing effective accessibility and network-mediated interactions. Weather spillovers are modeled using inverse-distance weights, reflecting the geographic propagation of meteorological processes. In the baseline specification, we use *K* = 5 nearest neighbors, balancing local sensitivity with robustness in sparsely populated high-altitude regions. Re-estimating all models *K* values from 4 to 10 yields highly stable results, with unchanged coefficient signs, magnitudes, and statistical significance (Supplementary Table 6).

We further assess residual spatial dependence by computing Moran’s I statistics on generalized residuals from the estimated models. Post-estimation Moran’s I values are substantially lower than their pre-estimation counterparts across all specifications. While some residual spatial correlation remains—most notably for international mobility—it is markedly attenuated, indicating that the model captures a substantial share of underlying spatial dependence.

Although the multinomial logit model relies on the Independence of Irrelevant Alternatives (IIA) assumption, the inclusion of spatially structured covariates allows substitution patterns to vary systematically with local and neighboring conditions. To assess the robustness of this assumption, we conduct drop-one-alternative tests, sequentially excluding each mobility category and re-estimating the model. Relative changes in coefficient vectors are generally modest and well below levels associated with substantive instability (Supplementary Table 7). Importantly, the direction and relative magnitude of key coefficients remain stable across all specifications.

While international labor migration—primarily to Russia and Kazakhstan—accounts for a large share of mobility in Kyrgyzstan, spatial spillovers remain relevant because domestic mobility, international mobility, and immobility are jointly determined within households and often reflect diversification strategies. Migration networks, credit constraints, and access to information are spatially clustered, particularly in remote and high-altitude areas. More broadly, the observed spatial correlation should not be interpreted solely as evidence of direct spillovers across districts. In mountainous settings such as Kyrgyzstan, neighboring districts often experience correlated climatic conditions, so part of the association likely reflects shared regional exposure rather than behavioral diffusion alone. These patterns are consistent with spatially clustered climatic stress being associated with local opportunity structures rather than direct behavioral diffusion across districts.

Finally, we assess whether overlapping weather extremes introduce confounding or multicollinearity. Pairwise correlations among dry spells, excessive rains, cold winters, and hot summers are modest ( | ρ | ≤ 0.196; Supplementary Table 8). Hot summers are the most prevalent extreme, followed by excessive rains, dry spells, and cold winters. While 26% of observations experience more than one extreme within a year, exposure to three or more events is rare, and four-way overlap is negligible. Consistent with these patterns, variance inflation factors remain close to unity for all climate variables (VIF ≤ 1.09; Supplementary Table 9), indicating that overlapping extremes do not bias the estimated weather effects.

### Reporting summary

Further information on research design is available in the Nature Portfolio Reporting Summary linked to this article.

## Supplementary information

Supplementary Information

Reporting Summary

Transparent Peer Review file

## Data Availability

The cleaned and operationalized dataset generated in this study has been deposited in the Zenodo repository^62^ under accession 10.5281/zenodo.20126869. Climate data are publicly available from the ERA5-Land reanalysis product of the European Centre for Medium-Range Weather Forecasts (ECMWF) at 10.24381/cds.e2161bac. The original Kyrgyz Integrated Household Survey (KIHS) microdata used in this study are subject to confidentiality and data-use restrictions imposed by the National Statistical Committee of the Kyrgyz Republic and therefore cannot be shared publicly by the authors. Researchers may request access to the original survey data from the National Statistical Committee of the Kyrgyz Republic (Marketing@stat.kg), subject to institutional approval and applicable data-use agreements.

## Code availability

Custom Python scripts used for data cleaning, spatial processing, estimation of the augmented multinomial logit (MNL) models, and calculation of marginal effects have been deposited in a public Zenodo^62^ repository under accession 10.5281/zenodo.20126869. The repository includes the analysis workflow, source code, and package requirements necessary to reproduce the analyses reported in this study. An executable reproducibility capsule containing the computational environment, code, and data workflow is additionally available on Code Ocean^63^ at 10.24433/CO.3566282.v1.

## References

[1] Linke, A. et al. Dry growing seasons predicted Central American migration to the US from 2012 to 2018. *Sci. Rep.* **13**, 18400 (2023). 37884560 10.1038/s41598-023-43668-9 PMC10603058

[2] Dinc, P. & Eklund, L. Syrian farmers in the midst of drought and conflict: the causes, patterns, and aftermath of land abandonment and migration. *Clim. Dev.* **16**, 349–362 (2024).

[3] Quiñones, E. J., Liebenehm, S. & Sharma, R. Left home high and dry-reduced migration in response to repeated droughts in Thailand and Vietnam. *Popul. Environ.* **42**, 579–621 (2021).

[4] Flood-induced selective migration patterns examined. *Nat. Clim. Change* **15**, 593-594 (2025). 10.1038/s41558-025-02346-6

[5] Wang, N. et al. Flood fatalities and displacement influence human migration in floodplains of developing countries. *Commun. Earth Environ.* **6**, 319 (2025).

[6] Roeckert, J. & Kraehnert, K. Extreme weather events and internal migration: evidence from Mongolia. *Econ. Disasters Clim. Change* **6**, 95–128 (2022).

[7] Upadhyay, H. Migration as good, bad and necessary: examining impacts of migration on staying Himalayan communities affected by climate change. *Humanit. Soc. Sci. Commun.* **11**, 1696 (2024).

[8] Ivanova, A. et al. Climate hazards and human migration: literature review. *Environ. Res. Clim.* **3**, 042002 (2024).

[9] Mallick, B., Priovashini, C. & Schanze, J. “I can migrate, but why should I?”—voluntary non-migration despite creeping environmental risks. *Humanit. Soc. Sci. Commun.* **10**, 1–14 (2023).

[10] Swapan, M. S. H. & Sadeque, S. Place attachment in natural hazard-prone areas and decision to relocate: research review and agenda for developing countries. *Int. J. Disaster Risk Reduct.* **52**, 101937 (2021).

[11] Benveniste, H., Oppenheimer, M. & Fleurbaey, M. Climate change increases resource-constrained international immobility. *Nat. Clim. Change* **12**, 634–641 (2022).

[12] Rikani, A., Otto, C., Levermann, A. & Schewe, J. More people too poor to move: divergent effects of climate change on global migration patterns. *Environ. Res. Lett.* **18**, 024006 (2023).

[13] Ayeb-Karlsson, S., Kniveton, D. & Cannon, T. Trapped in the prison of the mind: notions of climate-induced (im) mobility decision-making and wellbeing from an urban informal settlement in Bangladesh. *Palgrave Commun.* **6**, 1–15 (2020).

[14] Best, K., Gilligan, J. & Mallick, B. Economic inequality is a crucial determinant of observed patterns of environmental migration. *Commun. Earth Environ.* **6**, 1–8 (2025).39830897

[15] Betz, J. Migration. *Dev. Policy*, 181-188. 10.1007/978-3-658-35011-6_18 (2022).

[16] Blumenstock, J. E., Chi, G. & Tan, X. Migration and the value of social networks. *Rev. Econ. Stud.* **92**, 97–128 (2025).

[17] Jochim, V. & Macková, L. ‘I found everything in them’: formation of migrant networks and social capital. *Int. Migr.* **62**, 140–157 (2024).

[18] Sargent, K. Unpacking migration costs: heterogeneous effects in EU labor markets. *Econ. Model.* **139**, 106816 (2024).

[19] Almulhim, A. I. et al. Climate-induced migration in the Global South: an in depth analysis. *npj Clim. Action* **3**, 47 (2024).

[20] Kraemer, M. U. et al. Mapping global variation in human mobility. *Nat. Hum. Behav.* **4**, 800–810 (2020). 32424257 10.1038/s41562-020-0875-0

[21] Hoffmann, R., Šedová, B. & Vinke, K. Improving the evidence base: a methodological review of the quantitative climate migration literature. *Glob. Environ. Change* **71**, 102367 (2021).

[22] Duijndam, S. J. et al. Global determinants of coastal migration under climate change. *Nat. Commun.* **16**, 6866 (2025). 40715055 10.1038/s41467-025-59199-y PMC12297289

[23] Trinh, T.-A., Smyth, R., Churchill, S. A. & Yew, S. L. A financial disaster in the making: temperature shocks, climate change and savings. *Energy Res. Soc. Sci.* **118**, 103782 (2024).

[24] Bressan, G., Đuranović, A., Monasterolo, I. & Battiston, S. Asset-level assessment of climate physical risk matters for adaptation finance. *Nat. Commun.* **15**, 5371 (2024). 38951521 10.1038/s41467-024-48820-1 PMC11217445

[25] Aleksandrova, M. & Costella, C. Reaching the poorest and most vulnerable: addressing loss and damage through social protection. *Curr. Opin. Environ. Sustain.* **50**, 121–128 (2021).

[26] Gilmore, E. A. et al. Defining severe risks related to mobility from climate change. *Clim. Risk Manag.* **44**, 100601 (2024).

[27] Blondin, S. Staying despite disaster risks: place attachment, voluntary immobility and adaptation in Tajikistan’s Pamir Mountains. *Geoforum* **126**, 290–301 (2021).

[28] Sengupta, M. Environmental immobility: a systematic review of empirical research. *Ambio* **54**, 1729–1756 (2025). 40498236 10.1007/s13280-025-02195-9 PMC12480326

[29] Wiegel, H., Warner, J., Boas, I. & Lamers, M. Safe from what? Understanding environmental non-migration in Chilean Patagonia through ontological security and risk perceptions. *Reg. Environ. Change* **21**, 43 (2021).

[30] Pepin, N. C. et al. Climate changes and their elevational patterns in the mountains of the world. *Rev. Geophys.* **60**, e2020RG000730 (2022).

[31] Schneiderbauer, S. et al. Risk perception of climate change and natural hazards in global mountain regions: a critical review. *Sci. Total Environ.* **784**, 146957 (2021). 33895507 10.1016/j.scitotenv.2021.146957

[32] Wang, H. et al. Disaster effects of climate change in High Mountain Asia: state of art and scientific challenges. *Adv. Clim. Change Res.* **15**, 367–389 (2024).

[33] Saidaliyeva, Z. et al. Adaptation to climate change in the mountain regions of Central Asia: a systematic literature review. *Wiley Interdiscip. Rev. Clim. Change* **15**, e891 (2024).

[34] Adler, C. et al. In *Climate Change 2022: Impacts, Adaptation and Vulnerability*. *Contribution of Working Group II to the Sixth Assessment Report of the Intergovernmental Panel on Climate Change* (eds Pörtner, H.O. et al.) 2273–2318 (Cambridge University Press, 2022).

[35] Kumar, S. et al. Out-migration from the Indian Himalayan Region (IHR) with drivers, patterns and policy implications for sustainable mountain development. *Discov. Sustain.* **6**, 1381 (2025).

[36] Fallah, B., Didovets, I., Rostami, M. & Hamidi, M. Climate change impacts on Central Asia: trends, extremes and future projections. *Int. J. Climatol.* **44**, 3191–3213 (2024).

[37] Gummadi, S., Samineni, S. & Lopez-Lavalle, L. A. B. Assessing high-resolution precipitation extremes in Central Asia: evaluation and future projections. *Clim. Change* **178**, 29 (2025).

[38] Hermans, K. et al. Future research directions for understanding the interconnections between climate change, water scarcity, and mobility in rural Central Asia. *Clim. Dev.* **17**, 638–647 (2025).

[39] Sagynbekova, L. Environment, rural livelihoods, and labor migration: a case study in central Kyrgyzstan. *Mt. Res. Dev.* **37**, 456–463 (2017).

[40] McAuliffe, M., Coppari, P. R., Abbasi-Shavazi, M. J. & Maunganidze, O. In *World Migration Report* Vol. 2024 (eds McAuliffe, M. & Oucho, L.A.) e00037 (International Organization for Migration, 2024).

[41] Public Expenditure and Financial Accountability (PEFA). Kyrgyz Republic https://www.pefa.org/node/181 (2021).

[42] Schleicher, J. et al. Where nature and poverty meet: developing a multidimensional environment-poverty measure. * J. Dev. Stud.* **61**, 869–889 (2025).

[43] Rao, N. Migration, education and socio-economic mobility. *Comp. A J. Comp. Int. Educ.* **40**, 137–145 (2010).

[44] Adger, W. N., Barnett, J., Brown, K., Marshall, N. & O’brien, K. Cultural dimensions of climate change impacts and adaptation. *Nat. Clim. Change* **3**, 112–117 (2013).

[45] Kapruwan, R. et al. Climate change adaptation in global mountain regions requires a multi-sectoral approach. *Commun. Earth Environ.* **6**, 454 (2025).

[46] Kimsanova, B., Umirbekov, A., Herzfeld, T. & Müller, D. Heterogeneous effects of weather extremes on different dimensions of poverty in Kyrgyzstan. *Environ. Res. Lett.* **19**, 014068 (2024).

[47] Stark, O. & Bloom, D. E. The new economics of labor migration. * Am. Econ. Rev.* **75**, 173–178 (1985).

[48] Guriev, S. & Vakulenko, E. Breaking out of poverty traps: Internal migration and interregional convergence in Russia. *J. Comp. Econ.* **43**, 633–649 (2015).

[49] De Haas, H. A theory of migration: the aspirations-capabilities framework. *Comp. Migr. Stud.* **9**, 8 (2021). 33680858 10.1186/s40878-020-00210-4 PMC7902564

[50] Poletaev, D. V. Adaptation and integration of labour migrants from the Eaeu in Russia on the example of migrants from Kyrgyzstan. *Popul. Econ.* **4**, 20–37 (2020).

[51] Schewel, K. Understanding immobility: moving beyond the mobility bias in migration studies. *Int. Migr. Rev.* **54**, 328–355 (2020).

[52] Rigaud, K. K. et al. Groundswell: Preparing for Internal Climate Migration. *World Bank* **10**, 94 (2018).

[53] FAO & GCF. Social protection and inclusive climate action – A review of social protection for rural populations within Green Climate Fund projects. (FAO and Green Climate Fund, Rome and Incheon, 2025) https://www.greenclimate.fund/sites/default/files/document/20250918-social-protection-fao-joint-report.pdf.

[54] Muñoz-Sabater, J. et al. ERA5-Land: a state-of-the-art global reanalysis dataset for land applications. *Earth Syst. Sci. Data* **13**, 4349–4383 (2021).

[55] Conti, V., Sitko, N. J. & Ignaciuk, A. How do extreme weather events affect livestock herders’ welfare? Evidence from Kyrgyzstan. Report No. 18-07, 28 (FAO, 2018). https://openknowledge.fao.org/server/api/core/bitstreams/6bf23ad9-5933-4674-8a7c-c2fd69dce90b/content

[56] IPCC, 2023: Climate Change 2023: Synthesis Report. Contribution of Working Groups I, II and III to the Sixth Assessment Report of the Intergovernmental Panel on Climate Change [Core Writing Team, H. Lee and J. Romero (eds.)]. IPCC, Geneva, Switzerland, 184 pp., 10.59327/IPCC/AR6-9789291691647.

[57] Alkire, S. et al. *Multidimensional poverty measurement and analysis* (Oxford University Press, 2015).

[58] McKee, T. B., Doesken, N. J. & Kleist, J. In *8th Conference on Applied Climatology* 17, 179–183 (Anaheim, 1993).

[59] World Meteorological Organization, 2012: Standardized Precipitation Index User Guide (M. Svoboda, M. Hayes and D. Wood). (WMO-No. 1090), Geneva. https://www.droughtmanagement.info/literature/WMO_standardized_precipitation_index_user_guide_en_2012.pdf.

[60] Weiss, D. J. et al. Global maps of travel time to healthcare facilities. *Nat. Med.* **26**, 1835–1838 (2020). 32989313 10.1038/s41591-020-1059-1

[61] Körner, C., Paulsen, J. & Spehn, E. M. A definition of mountains and their bioclimatic belts for global comparisons of biodiversity data. *Alp. Bot.* **121**, 73–78 (2011).

[62] Kimsanova, B. et al. Household mobility responses to weather extremes in Kyrgyzstan (v1.0). *Zenodo.*10.5281/zenodo.20126870 (2026). 10.1038/s41467-026-75052-2 PMC13315783 42374037

[63] Kimsanova, B. et al. Household mobility responses to weather extremes – Analysis Code [Source Code]. 10.24433/CO.7128280.v2 (2026). 10.1038/s41467-026-75052-2 PMC13315783 42374037

