# Supplementary material for: Household mobility responses to weather extremes in Kyrgyzstan
Source: Nat Commun. 2026 Jun 29;17:5629. doi: 10.1038/s41467-026-75052-2 (PMC13315783; doi:10.1038/s41467-026-75052-2)
Supplement: Supplementary file 1 — Supplementary Information [file 41467_2026_75052_MOESM1_ESM.pdf]

Supplementary Information for:

Household mobility responses to weather extremes in Kyrgyzstan

Barchynai Kimsanova[1*], Thomas Herzfeld[1,2], Atabek Umirbekov[1], Kathleen Hermans[1], Daniel Müller[1,3,4], and Nodir Djanibekov[1]

[1]Leibniz Institute of Agricultural Development in Transition Economies (IAMO), Halle (Saale), Germany,
[2]Martin-Luther-Universität Halle-Wittenberg, Germany,
[3]Humboldt-Universität zu Berlin, Germany,
[4]Integrative Research Institute on Transformations of Human-Environment Systems (IRI THESys), Humboldt-Universität zu Berlin, Germany

[*]Corresponding author: Barchynai Kimsanova (kimsanova@iamo.de)

List of Supplementary Items

- Supplementary Note 1. Formal derivation of the resource-based mobility constraints model
- Supplementary Table 1. Panel structure of the KIHS, 2013–2022
- Supplementary Table 2. Construction of the Multidimensional Well-being Index (MWI)
- Supplementary Table 3. Definitions, measurement, units, and sources of variables
- Supplementary Methods 1. Spatial multinomial logit model specification
- Supplementary Table 4. Least squares estimate of the association between future migration and MWI
- Supplementary Table 5. Moran's I tests for spatial autocorrelation before model estimation
- Supplementary Table 6. Moran's I tests for spatial autocorrelation after model estimation
- Supplementary Table 7. Independence of Irrelevant Alternatives (IIA) diagnostics
- Supplementary Table 8. Overlap and dependence among weather extremes
- Supplementary Table 9. Variance inflation factors (VIF) for weather variables
- Supplementary References

Supplementary Note 1. Formal derivation of the resource-based mobility constraints model

We develop resource-based mobility constraints model that integrates the household strategy framework of the New Economics of Labour Migration (NELM)[1] with the spatial poverty trap model[2]. This synthesis extends existing theory by explicitly modeling domestic and international mobility as distinct but interlinked outcomes, each characterized by a different cost threshold. The framework is designed to capture both the push and constraining effects of weather extremes on household mobility outcomes.

Consider a household of size n, with members distributed across three locations: the origin (h), a domestic destination (d), and an international destination (f). Wages in these locations are denoted w_h, w_d, and w_f, respectively, with $w_h < w_d < w_f$. Let m_h, m_d, and m_f represent the number of household members residing at home, engaged in domestic mobility, and involved in international mobility. Mobility costs are denoted c_d for domestic mobility and c_f for international mobility, with $c_d < c_f$.

Household total income is given by

$$I = w_h m_h + w_d m_d + w_f m_f, \quad (1)$$

subject to the population constraint

$$n = m_h + m_d + m_f. \quad (2)$$

Household per capita utility as

$$U = \frac{I - c_d m_d - c_f m_f}{n}, \quad (3)$$

where mobility costs are deducted directly from household resources. The household chooses m_d and m_f to maximise U subject to these constraints.

The marginal utility of relocating one household member from the origin to a domestic and international destination is

$$\frac{\partial U}{\partial m_d} = \frac{w_d - w_h - c_d}{n}, \frac{\partial U}{\partial m_f} = \frac{w_f - w_h - c_f}{n}. \quad (4)$$

Domestic mobility occurs if

$$w_d - w_h - c_d > 0 \text{ and } w_d - w_h - c_d > w_f - w_h - c_f,$$

while international mobility is chosen if

$$w_f - w_h - c_f > 0 \text{ and } w_f - w_h - c_f > w_d - w_h - c_d.$$

If both net gains are non-positive ($w_d - w_h - c_d \leq 0$, $w_f - w_h - c_f \leq 0$), the household remains immobile, corresponding to a poverty trap.

Fig. 2 in the main text illustrates the resulting non-linear relationship between household resources, mobility costs, and mobility outcomes. The blue curve represents the probability of domestic mobility, which becomes positive once household resources exceed the domestic mobility cost threshold ($w_h > c_d$) and subsequently declines as international

mobility becomes feasible. The green curve represents the probability of international mobility, which emerges once household resources exceed the higher international mobility threshold ($w_h > c_f$). The shaded region corresponds to $w_h < c_d$, where mobility is infeasible, and vertical lines indicate the domestic and international mobility thresholds.

Extreme weather events enter the model as exogenous shocks to both wages and mobility costs. A push effect arises when climatic shocks reduce home earnings (w_h), increasing the relative gains from mobility and shifting both mobility curves upward. A constraining effect arises when extreme weather increases mobility costs (c_d, c_f), for example through asset losses, damaged infrastructure, or higher travel costs, shifting mobility curves downward or moving thresholds rightward. The net effect of climatic stress on mobility is therefore theoretically ambiguous, consistent with mixed findings in the empirical literature[3].

Supplementary Table 1 Panel structure of the KIHS, 2013–2022. Pairwise household retention rates (%) across survey waves. Each cell reports the percentage of households observed in the column year that are also observed in the row year. High retention between adjacent years and a gradual decline over longer intervals reflect the rotating panel design of KIHS rather than selective attrition.

	2013	2014	2015	2016	2017	2018	2019	2020	2021
2013									
2014	92								
2015	84	92							
2016	79	87	95						
2017	73	80	88	93					
2018	70	77	85	90	96				
2019	67	74	82	87	93	97			
2020	64	71	79	84	91	94	97		
2021	63	70	77	82	89	92	95	98	
2022	60	67	74	79	86	89	91	94	95

Supplementary Table 2 Construction of the Multidimensional Well-being Index (MWI). This table reports the dimensions, sub-dimensions, survey items, and weighting scheme used to construct the MWI. Nutrition is measured using a Berry Index of food diversity; health captures illness, access to healthcare, and the ability to cover medical expenses; education includes preschool attendance and educational attainment; and living standards are measured through income, living space, land, and livestock indicators. All components are standardized and normalized before aggregation.

Dimension	Sub-dimension	Explanation of sub-dimensions	Survey question	Weights
Nutrition	Berry Index	Food diversity is measured by Berry Index as follows: $$BI = 1 - \sum_{i=1}^{10} \omega_i^2,$$ where ω_i is the share of expenditures on food group i in the household's total consumption expenditure.	What kind of food products were consumed by members of your household during the surveyed 14 days?	Berry Index/4
Health	Illness	Identify the need for medical assistance and determine the reason for refusing medical services. We considered it poor if a family could not pay for medical care or buy medicine due to a lack of money.	Have you needed medical assistance in the past year? If yes ⇒ were there any cases during the year when you could not use medical services? If yes ⇒ for what reason did you not use medical services during the year?	Illness/12
	Healthcare	Identification of the need for inpatient treatment and determination of the reasons for refusal of inpatient treatment. We considered it poor if a family could not pay for hospital treatment due to a lack of money.	Have you been referred to a hospital or needed hospital treatment but did not go to the hospital in the past year? If, Yes, I was referred but did not go to the hospital, or/and Yes, I needed hospital treatment but didn't go to the hospital. ⇒ Reasons why you did not go to the hospital.	Healthcare/12
	Coverage of medical expenses	Identification of financial difficulties in covering medical expenses.	What did you have to do to use medical services over the past year?	Coverage of medical expenses/12
Education	Pre-school attendance	Identification of children who do not attend preschool and whether	Does (NAME) attend preschool? If no ⇒ what is the reason (NAME) is	Pre-school attendance/8

		this is due to financial difficulties.	not attending preschool?	
	Level of education	Identification of the household head's level of education and consider uneducated if the household head does not have 11 years of primary education.	What is the highest level of education you have received?	Level of education/8
Living Standard	Urban income<br>Rural income	The total household income (urban and rural) is calculated as the sum of recorded and deflated individual earnings that are aggregated into three main groups: wages, social transfers, and remittances. Values are standardized by the following formula: $z_i = \frac{x_i - \mu}{\sigma}$, then normalized by $n_i = \frac{x_i - \min(x)}{\max(x) - \min(x)}$.	What income did you receive over the past month and the amount of this income?	Income/16
	Urban living space<br>Rural living space	The living space available to each household member is calculated as the ratio of the living space to the number of household members. The obtained values are also standardized and normalized.	What is your family's living space (sq. m.)?	Living space/16
	Rural land	Amount of land owned by rural households, which is also standardized and normalized.	Do you have any land in use? If yes ⇒ what is the plots' total area (with the house) (sq. m.)?	Land/16
	Rural livestock	Livestock from various species is unified by the Livestock unit https://ec.europa.eu/eurostat/statistics-explained/index.php?title=Glossary:Livestock_unit_(LSU) and multiplied by the amount of livestock, then normalized and standardized.	What livestock, poultry, or other animals do you have?	Livestock/16

Supplementary Table 3 Definitions, measurement, units, and sources of variables used in the analysis. Descriptions, measurement approaches, coding schemes, and data sources for all variables included in the spatial multinomial logit models of household mobility outcomes in Kyrgyzstan, 2013–2022.

Variable	Description	Measurement	Unit	Source
Mobility	Household mobility outcome in the past 12 months	Change in household size from member relocation between $t-1$ to t; no change = immobility	1=Domestic 2=International 3=Combined 4=Immobility	KIHS
MWI	Multidimensional measure of household well-being	Composite index of nutrition, education, health, and living standards)	Continuous, 0=lowest well-being, 1=highest well-being	KIHS
Dry spells	Household exposure to drought conditions	Standardized Precipitation Index (SPI) over the hydrological year, aggregated to province level.	Thresholded SPI (continuous; values ≤ −1 retained, others set to zero)	ERA5
Excessive rains	Household exposure to heavy rainfall or flooding	Monthly SPI in spring, aggregated to district level	Thresholded SPI (continuous; values > 1 retained, others set to zero)	ERA5
Hot summers	Household exposure to extreme heat	Monthly Standardized Temperature Index (STI) for July-August, aggregated to district level; continuous values retained, with values ≤ 1 set to zero	Thresholded STI (continuous; values > 1 retained, others set to zero)	ERA5
Cold winters	Household exposure to extreme cold	Monthly STI for January-March, aggregated to district level; continuous values retained, with values ≥ −1 set to zero	Thresholded STI (continuous; values < −1 retained, others set to zero)	ERA5

Rural households	Household located in a rural area	Classified based on geographic location	Binary (1 = Rural, 0 = Urban)	KIHS
Household size	Household size	Total number of individuals residing in the household	Count (persons)	KIHS
Dependency ratio	Household demographic dependency burden	Ratio of dependents (children and elderly) to working-age household members	Continuous (ratio)	KIHS
Male share	Gender composition of the household	Share of male members in total household size	Continuous (0–1)	KIHS
Elevation thresholds	Household altitude category	Three alternative binary indicators for households located above 1,000 m, 1,500 m, and 2,000 m.a.s.l. (applied in separate specifications)	Binary (1 = above threshold, 0 = otherwise)	DEM

Supplementary Methods 1 Spatial multinomial logit model specification

Our model evaluates how household well-being (MWI) and extreme weather events influence mobility outcomes across lower-, middle-, and higher-well-being groups, defined by terciles of the MWI distribution, allowing us to examine potential non-linear relationships between well-being and mobility under varying climatic conditions. The dependent variable is categorical and captures four mutually exclusive mobility outcomes observed over the previous 12 months: domestic mobility ($j = 1$), international mobility ($j = 2$), combined mobility ($j = 3$), with immobility ($j = 4$) serving as the reference category.

The model is specified as:

$$\ln\left(\frac{P(Y=j)}{P(Y=\text{immobility})}\right) = \alpha_j^\tau + \beta^\tau MWI_{i,t-1} + \boldsymbol{\gamma}^\tau \boldsymbol{C}_{i,t} + \boldsymbol{\delta}^\tau \boldsymbol{H}_{i,t} + \boldsymbol{\varphi}^\tau \boldsymbol{I}_{i,t} + \boldsymbol{\theta}^\tau W_i \boldsymbol{X}_{i,t} \quad (5)$$

where $P(Y = j)$ denotes the probability of household i experiencing mobility outcome j relative to immobility. MWI_{t-1} is the lagged MWI, capturing household conditions in the previous year to reduce simultaneity bias. Vector $\boldsymbol{C}_{i,t}$ includes climate exposure variables capturing dry spells, excessive rainfall, cold winters, and hot summers. Vector $\boldsymbol{H}_{i,t}$ contains household characteristics, including rural residence, household size, dependency ratio, and male share.

Vector $\boldsymbol{I}_{i,t}$ captures interaction terms between elevation and key local covariates (MWI, household characteristics, and weather extremes). Elevation is operationalized using alternative binary indicators for households located above 1,000 m, 1,500 m, and 2,000 m above sea level, which are introduced in separate specifications.

Spatial dependence is incorporated through spatially weighted neighboring covariates $W_i\boldsymbol{X}_{i,t}$, which represents spatial lags of selected explanatory variables. These terms capture spillover effects in household well-being and climate exposure across neighboring districts. Separate spatial weighting matrices are used for household and climate variables. Household spillovers are constructed using a travel-cost-based spatial matrix, while climate spillovers rely on inverse-distance relationships based on geographic proximity. Spatial weights matrices are constructed using a K-nearest neighbors approach with $K = 5$.

All coefficients α_j^τ, β^τ, $\boldsymbol{\gamma}^\tau$, $\boldsymbol{\delta}^\tau$, $\boldsymbol{\lambda}^\tau$, and $\boldsymbol{\theta}^\tau$ are estimated separately for each MWI tercile group τ. Inference is based on a primary sampling unit (PSU) cluster bootstrap, and results are interpreted using average marginal effects.

Supplementary Table 4 Least squares estimate of the association between future migration and MWI. This table reports weighted least squares (WLS) estimates of the relationship between future household migration and the MWI. Column (1) presents a baseline specification including future migration only, while Column (2) adds household composition controls, elevation, and year fixed effects. All models use survey weights, and standard errors are clustered at the primary sampling unit (PSU) level. Diagnostic statistics are reported for completeness.

**Dep. var.: MWI**	**Baseline model**		**Extended controls**	
	Coef.	St. err.	Coef.	St. err.
Future migration	-.0063	.003	-.0090	.003
Dependency ratio			-.0362	.004
Household size			-.0046	.001
Male share			.0013	.005
Elevation (m.a.s.l.)			-1.074e-05	1.78e-06
Year fixed effects	No		Yes	
R-squared	.001		.175	
Observations	40142		40139	
Durbin-Watson	.878		.864	
Jarque-Bera	264326.529		132890.277	
Skewness	-2.460		-1.784	
Kurtosis	14.568		11.169	

Supplementary Table 5 Moran's I tests for spatial autocorrelation before model estimation. This table reports Moran's I statistics computed prior to model estimation for weather variables and mobility outcomes using k-nearest neighbor spatial weights (k = 4–10). All variables exhibit statistically significant positive spatial autocorrelation ($p < 0.001$), motivating the inclusion of spatial lags in the regression models.

Variable category	Variables	Moran's I (range)	Neighborhoods (k)	p-value
Weather	Dry spells, Excessive rains, Hot summers, Cold winters	.54-.91	4-10	<0.001
Mobility	Mobility outcome	.51-.57	4-10	<0.001

Supplementary Table 6 Moran's I tests for spatial autocorrelation after model estimation. This table reports Moran's I statistics computed on generalized residuals from the spatial multinomial logit model using k-nearest neighbor spatial weights ($k = 5$). Results are aggregated across MWI terciles and elevation thresholds. Compared to pre-estimation diagnostics (Tab. S9), spatial autocorrelation is substantially reduced after accounting for spatial lags and covariates, indicating that the model adequately captures spatial dependence in mobility outcomes.

Mobility outcome	Mean Moran's I	Min	Max	Share significant
Domestic	.128	.073	.205	.75
International	.319	.140	.423	1.00
Combined	.123	.077	.206	.75
Immobility	.317	.134	.422	1.00

Supplementary Table 7 Independence of Irrelevant Alternatives (IIA) diagnostics. This table summarizes across MWI tercile groups and elevation thresholds. Reported values describe the relative change in coefficient vectors (L2 norm) when one mobility alternative is removed from the choice set. "Mean," "Max," and "Min change" refer to relative coefficient changes across tests; each specification is based on six drop-one-alternative tests, corresponding to all feasible alternative removals. "Share > 10" and "Share > 20" indicate the proportion of tests in which relative coefficient changes exceed 0.10 and 0.20, respectively. "Pot. sen." denotes potential sensitivity, flagging specifications with comparatively larger maximum coefficient changes.

Tercile	Elevation	Mean change	Max change	Min change	N (full model)	Share > 10	Share > 20	IIA flag
Lower	None	.072	.192	.018	13197	.167	0	OK
Lower	>1000	.093	.142	.049	13197	.333	0	OK
Lower	>1500	.107	.294	.026	13197	.333	.167	OK
Lower	>2000	.198	.480	.060	13197	.500	.333	OK
Middle	None	.101	.189	.047	13227	.500	0	OK
Middle	>1000	.213	.422	.067	13227	.833	.500	OK
Middle	>1500	.283	.470	.132	13227	1	.500	OK
Middle	>2000	.178	.288	.128	13227	1	.333	OK
Higher	None	.589	.977	.053	13715	.833	.667	Pot. sen
Higher	>1000	.590	.966	.310	13715	1	1	Pot. sen
Higher	>1500	.478	.928	.118	13715	1	.667	Pot. sen
Higher	>2000	.414	.867	.190	13715	1	.667	Pot. sen

Supplementary Table 8 Overlap and dependence among weather extremes. This table reports pairwise correlations and co-occurrence rates of weather extremes used in the analysis (N = 47,112). While concurrent extreme events are common, pairwise correlations remain modest, indicating limited linear dependence among weather variables.

Variable pair / statistic	Value
**Pairwise correlations**	
Dry spells x Excessive rains	.026
Dry spells x Cold winters	.006
Dry spells x Hot summers	.089
Excessive rains x Cold winters	.002
Excessive rains x Hot summers	.146
Cold winters x Hot summers	.028
**Event prevalence**	
Dry spells	.170
Excessive rains	.303
Cold winters	.098
Hot summers	.495
**Concurrent exposures**	
Two or more concurrent weather exposures	.264
Three or more concurrent weather exposures	.016

Four concurrent exposures	.0002

Supplementary Table 9 Variance inflation factors (VIF) for weather variables. Variance inflation factors are computed from auxiliary regressions using the full analysis sample (N = 47,112). All VIF values are well below conventional thresholds, indicating no meaningful multicollinearity despite partial overlap of weather extremes.

**Variable**	**R^2 (auxiliary)**	**VIF**
Dry spells	.055	1.06
Excessive rains	.081	1.09
Cold winters	.081	1.09
Hot summers	.030	1.03

Supplementary References

1 Stark, O. & Bloom, D. E. The new economics of labor migration. *The american Economic review* **75**, 173-178 (1985).

2 Guriev, S. & Vakulenko, E. Breaking out of poverty traps: Internal migration and interregional convergence in Russia. *Journal of comparative economics* **43**, 633-649 (2015).

3 Kaczan, D. J. & Orgill-Meyer, J. The impact of climate change on migration: a synthesis of recent empirical insights. *Climatic change* **158**, 281-300 (2020).
