# Supplementary material for: Household mobility responses to weather extremes in Kyrgyzstan
Source: Nat Commun. 2026 Jun 29;17:5629. doi: 10.1038/s41467-026-75052-2 (PMC13315783; doi:10.1038/s41467-026-75052-2)
Supplement: Supplementary file 2 — Reporting Summary [file 41467_2026_75052_MOESM2_ESM.pdf]

nature portfolio

Corresponding author(s): Barchynai Kimsanova

Last updated by author(s): May 8, 2026

Reporting Summary

Nature Portfolio wishes to improve the reproducibility of the work that we publish. This form provides structure for consistency and transparency in reporting. For further information on Nature Portfolio policies, see our Editorial Policies and the Editorial Policy Checklist.

Statistics

For all statistical analyses, confirm that the following items are present in the figure legend, table legend, main text, or Methods section.

n/a	Confirmed	
☐	☒	The exact sample size (*n*) for each experimental group/condition, given as a discrete number and unit of measurement
☐	☒	A statement on whether measurements were taken from distinct samples or whether the same sample was measured repeatedly
☐	☒	The statistical test(s) used AND whether they are one- or two-sided<br>*Only common tests should be described solely by name; describe more complex techniques in the Methods section.*
☐	☒	A description of all covariates tested
☐	☒	A description of any assumptions or corrections, such as tests of normality and adjustment for multiple comparisons
☐	☒	A full description of the statistical parameters including central tendency (e.g. means) or other basic estimates (e.g. regression coefficient) AND variation (e.g. standard deviation) or associated estimates of uncertainty (e.g. confidence intervals)
☐	☒	For null hypothesis testing, the test statistic (e.g. *F*, *t*, *r*) with confidence intervals, effect sizes, degrees of freedom and *P* value noted<br>*Give P values as exact values whenever suitable.*
☒	☐	For Bayesian analysis, information on the choice of priors and Markov chain Monte Carlo settings
☐	☒	For hierarchical and complex designs, identification of the appropriate level for tests and full reporting of outcomes
☐	☒	Estimates of effect sizes (e.g. Cohen's *d*, Pearson's *r*), indicating how they were calculated

Our web collection on statistics for biologists contains articles on many of the points above.

Software and code

Policy information about availability of computer code

Data collection	No commercial or proprietary software was used for data collection. Household survey and climate data were obtained from official sources. Data cleaning, merging, spatial processing, and statistical analyses were conducted in Python (version 3.11) using open-source econometric and spatial analysis libraries. Custom scripts were developed to integrate survey and climate datasets, estimate the augmented multinomial logit (MNL) models, perform PSU-cluster bootstrap inference, and compute average marginal effects. The analysis code is available from the authors upon reasonable request.
Data analysis	All analyses were conducted in Python (version 3.11) using open-source libraries. Data management and preprocessing were performed with pandas (v2.2.2) and numpy (v1.26.4). Econometric estimation and marginal effects calculations were implemented using statsmodels (v0.14.2), while supplementary statistical procedures relied on scikit-learn (v1.5.1). Spatial dependence and spatial weights matrices were constructed using libpysal (v4.10.0). Custom Python scripts were developed to integrate household survey and climate datasets, estimate the augmented multinomial logit (MNL) models, perform PSU-cluster bootstrap inference, and compute average marginal effects. No commercial or proprietary software was used. The custom analysis code will be made publicly available through Code Ocean upon publication.

For manuscripts utilizing custom algorithms or software that are central to the research but not yet described in published literature, software must be made available to editors and reviewers. We strongly encourage code deposition in a community repository (e.g. GitHub). See the Nature Portfolio guidelines for submitting code & software for further information.

Data

Policy information about availability of data

All manuscripts must include a data availability statement. This statement should provide the following information, where applicable:

- Accession codes, unique identifiers, or web links for publicly available datasets
- A description of any restrictions on data availability
- For clinical datasets or third party data, please ensure that the statement adheres to our policy

The climate data used in this study are publicly available from the ERA5-Land reanalysis product of the European Centre for Medium-Range Weather Forecasts (ECMWF) (https://doi.org/10.24381/cds.e2161bac). The household survey data (Kyrgyz Integrated Household Survey, KIHS) were obtained from the National Statistical Committee of the Kyrgyz Republic under a purchase agreement. Due to licensing and confidentiality restrictions, these household data cannot be shared by the authors. Researchers may obtain access directly from the National Statistical Committee of the Kyrgyz Republic upon request (https://stat.kg/en/). Custom analysis code and processed data required to reproduce the analyses will be made publicly available through Code Ocean upon publication.

Research involving human participants, their data, or biological material

Policy information about studies with human participants or human data. See also policy information about sex, gender (identity/presentation), and sexual orientation and race, ethnicity and racism.

Reporting on sex and gender	This study analyzes household-level mobility outcomes rather than individual-level behavior. The household survey (KIHS) includes demographic characteristics of household members, and the analysis incorporates an aggregate household-level measure of sex composition (male share) as a control variable. However, the study does not investigate sex- or gender-specific mobility behavior, and no analyses were stratified or interpreted by sex or gender. Sex and gender were therefore not primary considerations in the study design, as the research objective was to examine how household well-being and weather extremes shape household mobility outcomes. The findings are thus interpreted at the household level rather than the individual level. Sex-related information in the underlying survey was self-reported by respondents. No sex- or gender-disaggregated results are presented.
Reporting on race, ethnicity, or other socially relevant groupings	This study did not include race or ethnicity as analytical variables. Instead, the analysis focused on socially relevant household and geographic groupings, including household well-being terciles (low-, middle-, and high-) derived from a multidimensional well-being index, rural versus urban residence, and elevation-based classifications of districts. These groupings were selected because they are directly relevant to household mobility decisions and exposure to weather extremes in Kyrgyzstan. All classifications were based on self-reported survey information or administrative geographic data. Potential confounding was addressed by including household demographic, socioeconomic, climatic, and spatial control variables in the augmented multinomial logit models, with additional robustness analyses reported in the Supplementary information.
Population characteristics	The analysis uses household-level data from the nationally representative Kyrgyz Integrated Household Survey (KIHS). The unit of analysis is the household rather than individual participants. Household observations span multiple survey years and include information on socioeconomic conditions, demographic composition, and geographic characteristics relevant to mobility decisions and climate exposure.
Recruitment	Household data were obtained from the nationally representative Kyrgyz Integrated Household Survey (KIHS), administered by the National Statistical Committee of the Kyrgyz Republic using stratified random sampling procedures. The survey is designed to ensure national and regional representativeness. As with many household surveys, highly mobile households may be somewhat underrepresented because of temporary absence or non-response; however, the large sample size and survey design help reduce the risk of substantial sampling bias.
Ethics oversight	This study relied exclusively on secondary anonymized data from the Kyrgyz Integrated Household Survey (KIHS), which is administered by the National Statistical Committee of the Kyrgyz Republic in accordance with national statistical and data protection regulations. The authors did not collect primary human subject data, and no personally identifiable information was accessed. Therefore, no additional institutional ethical approval was required for this secondary data analysis.

Note that full information on the approval of the study protocol must also be provided in the manuscript.

Field-specific reporting

Please select the one below that is the best fit for your research. If you are not sure, read the appropriate sections before making your selection.

☐ Life sciences ☒ Behavioural & social sciences ☐ Ecological, evolutionary & environmental sciences

For a reference copy of the document with all sections, see nature.com/documents/nr-reporting-summary-flat.pdf

Behavioural & social sciences study design

All studies must disclose on these points even when the disclosure is negative.

Study description	This study is a quantitative observational analysis based on secondary household survey data from the Kyrgyz Integrated Household Survey (KIHS, 2013–2022), linked with gridded climate data from the ERA5-Land reanalysis product. The analysis examines how household well-being, weather extremes, and geographic context are associated with different household mobility outcomes in

	Kyrgyzstan using augmented multinomial logit models with spatial spillover components.
Research sample	The research sample consisted of household-year observations from the nationally representative Kyrgyz Integrated Household Survey (KIHS) spanning 2013–2022. The analytical sample included 40,150 household-year observations across four elevation categories (<1000 m, 1000–1500 m, 1500–2000 m, and >2000 m above sea level), corresponding to 6,295 unique households. The sample included information on mobility outcomes, multidimensional well-being, household demographic characteristics, and exposure to extreme weather events. Analyses were conducted at the household level and stratified by household well-being terciles.
Sampling strategy	The study uses data from the nationally representative Kyrgyz Integrated Household Survey (KIHS), a quarterly rotating panel covering approximately 5,000 households annually across all provinces of Kyrgyzstan and Bishkek during 2013–2022. The KIHS employs stratified random sampling to ensure representativeness across regions and between rural and urban areas. The analysis relies on anonymized secondary survey data linked with district-level climate information from the ERA5-Land reanalysis product. After excluding observations lacking geographic identifiers, the analytical sample retained approximately 97% of surveyed households and included repeated household observations over time, enabling robust longitudinal analysis of mobility, household well-being, and weather extremes.
Data collection	This study relies exclusively on secondary data sources. Household-level information was obtained from the Kyrgyz Integrated Household Survey (KIHS), administered annually by the National Statistical Committee of the Kyrgyz Republic using standardized interviewer-led questionnaires. Data collection was conducted by trained enumerators during household visits following official survey protocols. The authors were not involved in primary data collection and had no influence on how the raw survey data were recorded. Climate data were obtained independently from the publicly available ERA5-Land reanalysis product, which combines observational and model-based climate information.
Timing	The Kyrgyz Integrated Household Survey (KIHS) is conducted quarterly by the National Statistical Committee of the Kyrgyz Republic. Although household information is collected quarterly, migration outcomes are recorded annually; accordingly, this study uses annual household mobility observations covering the period 2013–2022, with approximately 5,000 households surveyed per year. Climate data were obtained from the ERA5-Land reanalysis product using monthly temperature and precipitation estimates at approximately 9 km spatial resolution. Standardized climate indices were constructed using the full historical ERA5-Land record, while the analytical period was restricted to 2013–2022 to align with the household survey data.
Data exclusions	Approximately 3% of surveyed household observations were excluded because they lacked valid district-level geographic identifiers and therefore could not be linked to spatial climate and geographic covariates. This exclusion criterion was defined prior to analysis. Additional exclusions occurred only where observations contained missing values for variables required in specific model specifications.
Non-participation	This study relies on secondary data from the Kyrgyz Integrated Household Survey (KIHS). Participant recruitment, response rates, and attrition were managed by the National Statistical Committee of the Kyrgyz Republic and are not directly observable to secondary data users. The analysis includes all household observations with valid geographic identifiers and complete information for the variables required in the respective model specifications.
Randomization	This study is observational and did not involve experimental assignment or randomization. Households were classified into well-being terciles (low-, middle-, high-) based on their Multidimensional Well-being Index (MWI) scores derived from survey data. Because group membership was not randomly assigned, the analyses controlled for potential confounding factors, including household demographic characteristics, rural versus urban location, elevation, and exposure to extreme weather events. Additional robustness checks are reported in the Supplementary information.

Reporting for specific materials, systems and methods

We require information from authors about some types of materials, experimental systems and methods used in many studies. Here, indicate whether each material, system or method listed is relevant to your study. If you are not sure if a list item applies to your research, read the appropriate section before selecting a response.

Materials & experimental systems

n/a	Involved in the study
☒	☐ Antibodies
☒	☐ Eukaryotic cell lines
☒	☐ Palaeontology and archaeology
☒	☐ Animals and other organisms
☒	☐ Clinical data
☒	☐ Dual use research of concern
☒	☐ Plants

Methods

n/a	Involved in the study
☒	☐ ChIP-seq
☒	☐ Flow cytometry
☒	☐ MRI-based neuroimaging

Plants

Seed stocks	n/a
Novel plant genotypes	n/a
Authentication	n/a
