# Supplementary material for: Household mobility responses to weather extremes in Kyrgyzstan
Source: Nat Commun. 2026 Jun 29;17:5629. doi: 10.1038/s41467-026-75052-2 (PMC13315783; doi:10.1038/s41467-026-75052-2)
Supplement: Supplementary file 3 — Transparent Peer Review file [file 41467_2026_75052_MOESM3_ESM.pdf]

nature portfolio

Peer Review File

Household mobility responses to weather extremes in Kyrgyzstan

Corresponding Author: Dr Barchynai Kimsanova

This file contains all reviewer reports in order by version, followed by all author rebuttals in order by version.

Version 0:

Reviewer comments:

Reviewer #1

(Remarks to the Author)
Dear Authors,

It has been a pleasure to review your manuscript “Household Mobility Responses to Weather Extremes.” The paper presents an interesting case examining the climate change–mobility nexus in a relatively understudied region. However, it requires substantial revisions to strengthen the robustness of the empirical approach and to develop a clearer identification strategy for causal inference, particularly considering the local context. Moreover, the paper’s contribution to the existing literature on climate–mobility interactions — including studies focusing on Central Asia and Kyrgyzstan — is not sufficiently articulated. The author should also provide a clearer description of the country case and discuss attrition patterns in the dataset over the study period (2013–2022).

Overall, the paper has significant potential for publication, but major revisions are necessary to enhance its methodological rigor and clarify its contribution.

Please find my detailed comments and suggestions for revision in the attached report.

With kind regards,
Reviewer

(Remarks on code availability)

Reviewer #2

(Remarks to the Author)
The paper is well-written, ambitious, and presents a novel wealth-based framework for understanding mobility under extreme weather in Kyrgyzstan. It combines household survey data with high-resolution climate information and a spatial econometric model, which is a strong methodological choice. However, several limitations undermine the strength of its claims. The paper tends to overstate causal inference, underexplains methodological decisions, and overlooks some crucial conceptual dimensions (e.g., social networks, governance, migration policies) that are essential to understanding mobility. This paper is innovative and empirically rich, but it currently risks overclaiming and oversimplifying. To achieve publication-level strength, it requires a more balanced conceptual framework, cautious causal language, stronger contextualization, and greater policy relevance. I hope the following remarks are helpful:
I was wondering if the framework is highly wealth-centric, with mobility outcomes explained almost exclusively through the household Multidimensional Well-being Index (MWI). While this is valuable, it risks reductionism. As there is now considerable evidence in the migration literature that suggests social networks, governance regimes, migration policies, and aspirations play a critical role, alongside wealth. It is expected to broaden the conceptual framing beyond wealth thresholds to include migration aspirations, social capital, cultural attachments, and institutional enablers/barriers. This would prevent over-simplification, perhaps by providing a conceptual diagram that integrates wealth with non-economic drivers of mobility (e.g., network effects, governance quality, migration regimes).
It implies the causal relationships between extreme weather events and mobility responses, yet the methodology (spatial

multinomial logit with lagged predictors) is correlational. But it cannot isolate causal mechanisms. So it is expected that the claims are associations rather than causal effects. If causal language is retained, justify it with robustness checks (such as instrumental variables, quasi-experiments, or longitudinal event-history models), and explicitly discuss concerns about endogeneity (e.g., mobility could affect well-being, not only the reverse).
The Multidimensional Well-being Index (MWI) is adapted, but choices such as replacing child mortality with dietary diversity are unjustified. Mobility data rely on annual household reports and exclude short-term, circular, or undocumented migration, which may bias results. Additionally, climate extremes are defined with strict cutoffs (SPI, STI), which may overlook localized hazards and perceptions. It requires sensitivity analyses showing how results change under alternative definitions of mobility and well-being, as well as how unobserved mobility (temporary or undocumented) could impact conclusions.
While the paper briefly references the Andes, Himalayas, and East Africa, this is superficial. It misses an opportunity to demonstrate the model's global applicability. It is possible to expand the comparative analysis—what lessons from Kyrgyzstan are generalizable to other mountainous regions, and what is unique to this case?
The recommendations (infrastructure, social protection, relocation assistance) are broad and could apply to almost any migration-climate paper. They don't directly emerge from the results. It is suggested to translate the findings into specific policy insights for Kyrgyzstan. For example, what role should Kyrgyz migration agreements with Russia or Kazakhstan play? How can social protection programs be tailored to mountainous regions? Or any other context-specific adaptation and migration policy suggestions that align with Kyrgyzstan's governance, institutions, and labor market realities.
Although the authors acknowledge but do not adequately analyze cultural ties, place attachment, and voluntary immobility. These are critical in mountain communities, where a strong attachment to ancestral land is prevalent. A subsection on voluntary immobility and its complications for the wealth-based threshold model would be beneficial for readers, which could be achieved by integrating qualitative evidence (from other studies, secondary data, or literature) on how immobility is not only poverty-driven but also culturally/psychologically rooted.
Minor Issues
• Writing sometimes overstates novelty: distinguishing four outcomes (immobile, domestic, international, combined) is useful, but not entirely new in migration studies.
• Figures (especially Fig. 1 & 5) need more clarity—threshold dynamics could be misread without stronger explanations.
• The robustness section is buried in methods; it should be summarized in results.

(Remarks on code availability)
I did not review the code

Reviewer #3

(Remarks to the Author)
The paper address an important question: how does poverty interact with mobility in response to weather extremes. The paper indicates for Kyrgyzstan how wealth influences mobility, which is likely to be an important question in other jurisdictions. Wealth thresholds are divided into 3 based on the multidimensional poverty index MPI.
Some literature on mobility is missing or not referenced.
'combined mobility' should be described early in the paper – domestic mobility is not clearly explained (see below)

Strong points:
The paper introduces a new spatial model that layers household panel data with high-resolution climate records and spatial modelling to assess how multidimensional well-being (MWI), extreme weather events, and geography influence mobility in Kyrgyzstan.
For Kyrgyzstan, the paper highlights how weather related impacts in nearby districts inhibit migration for both the wealthy and poor households. This is an interesting point, as it shows that apart from just the resources to migrate/be mobile, structures for safe mobility can promote or inhibit mobility.

Some issues to clarify earlier on:
Earlier on, where the authors tell us what the paper does, they should also tell us what it doesn't do. This comes later in Line 350 where the authors state that their model captures material and service-related dimensions but cannot fully reflect non-material factors such as social capital.
While the paper introduces a new model, it overlooks a foundational literature on immobility and mobility, particularly work by De Haas, H. (2021) A theory of migration: the aspirations-capabilities framework. CMS 9, 8 (2021). https://doi.org/10.1186/s40878-020-00210-4

The concept of 'combined migration' is difficult to understand and follow in the paper. This is particularly evident in a situation where the authors in one sentence say domestic mobility declined, while international mobility rose. In the next sentence, they argue that combined mobility became rare. So, the question becomes, "What is combined migration, and why does it matter?"
Include an overall statement about climate change in Kyrgyzstan

The term domestic mobility is unclear. L174 Does this mean the whole household moves?

L109 what about movement within households, where some family members move first.

L184 Entire households do not always move abroad, some members move first, some cannot move because of age, some send members abroad first to diversity incomes, and often male household members move first or children are sent to relatives. Even with the extreme weather events mentioned in the paper, does the data indicate whether the entire household unit moves? L253 does mention this but only in terms of sending one member abroad while others relocate locally.

Specific comments

Line 39 – apart from the costs of moving and adapting, what about access to relatives
Fig 1 explain what is meant by international threshold and international mobility. Whilst for Kyrgyzstan, it may mean neighbouring countries (Tajikistan, Uzbekistan, Kazakhstan…etc) but unclear does it mean beyond. More generally mobility across borders also depends on geography, borders (porous borders), and access. Although such information may not be available from the data set used.

L63 –'enabling wealthier households to move abroad outward…" why just abroad here and what about intrahousehold movement. Rarely to entire households move abroad at once.

L70 Another factor is the geographical location of the country and national borders

L82 Geographic barriers AND geopolitical borders – there is an assumption that even if you want to move across a border and have the wealth, you can move.

L191-193 makes an important point about regional shocks flattening differences in mobility across districts, which would indicate that domestic mobility is quite local or regional first?

L219 -221 first sentence -… "it does not capture the additional effects of compound extreme weather events.

L266 – 267 add at the end – of 'do not produce a uniform mobility outcome.. of household units? Or of households

Discussion and Concluding remarks
Might be good to mention planned relocation options? They exist in other countries, do they exist in Kyrgyzstan? Is it likely that the government will engage in planned relocation options in the future? if so why or why not?

(Remarks on code availability)

Version 1:

Reviewer comments:

Reviewer #1

(Remarks to the Author)
The authors have provided a response to the previous review and have implemented a number of revisions. The discussion of the spatial specification, the treatment of climate variables, and the description of the survey design are improved. The manuscript now offers clearer explanations of the empirical strategy, additional robustness checks, and improved explanation regarding the rotating panel structure of the KIHS dataset and the spatial modelling approach. These revisions improves the clarity and interpretability of the analysis.

However, a few aspects could still benefit from further clarification:
(i) The authors note that the empirical results should be interpreted as conditional associations rather than causal estimates. This clarification is welcome. Nonetheless, the manuscript could briefly acknowledge that lagging the well-being index may not fully eliminate potential feedback mechanisms between migration and household welfare, for example through remittances or labor reallocation.

(ii) The explanation of the spatial modelling framework is much clearer in the revised version, and the additional diagnostics are helpful. At the same time, it may be useful to acknowledge that some of the spatial correlation observed in the results may reflect shared regional exposure to climate conditions rather than direct spillover mechanisms across districts.

Recommendation: Accept after minor revisions.

(Remarks on code availability)

Reviewer #2

(Remarks to the Author)
The authors have responded carefully and constructively to the reviewer comments, and the revised manuscript is substantially improved as a result. The revised manuscript is substantially improved. The revisions demonstrate a thoughtful engagement with the critiques, and many of the earlier concerns have been addressed through clearer conceptual framing, more cautious interpretation of results, and improved transparency in the methodological description.
I found the authors have convincingly moderated the language around causal inference. The revised manuscript now consistently frames the results as conditional associations rather than causal effects, which is appropriate given the observational nature of the data and the spatial econometric approach used. The explicit discussion of endogeneity concerns and methodological limitations enhances the analytical credibility of the paper.
The authors have made a meaningful effort to broaden the conceptual framing beyond a purely wealth-centered interpretation. By situating the analysis within the aspirations–capabilities framework and acknowledging the role of social networks, governance structures, migration regimes, and cultural attachments, the manuscript now better reflects the complexity of migration decision-making. Although these factors cannot be directly modeled with the available data, the authors appropriately recognize their importance and clearly delimit the empirical scope of the study.
The manuscript now provides much clearer justification for methodological choices, including the construction of the Multidimensional Well-being Index (MWI), the treatment of climate indicators, and the specification of spatial weights. These revisions significantly improve the transparency and reproducibility of the study.
The discussion of mountain-region comparability has been expanded and now situates the Kyrgyzstan case more convincingly within the broader literature on mobility. This helps clarify which mechanisms may be generalizable (e.g., resource thresholds, spatial exposure, and mobility costs) and which remain context-specific.
Authors also carefully revised the policy implications section, which is much stronger and more contextually grounded. The authors now link their findings directly to Kyrgyzstan’s institutional context, including labor migration arrangements with Russia and Kazakhstan, spatially targeted social protection in mountain districts, and the need for coordinated adaptation planning across neighboring administrative units. These additions enhance the study's practical relevance.
In addition, the manuscript now more clearly addresses voluntary immobility and place attachment, acknowledging that immobility in mountain communities can also stem from cultural ties and identity-based connections to land. The clarification that the empirical framework focuses on capabilities rather than aspirations is an important conceptual improvement.
Overall, the revisions significantly strengthen the manuscript’s clarity, conceptual balance, and methodological transparency, while preserving its original empirical contribution. The study remains an ambitious and valuable contribution to the literature on climate-related mobility, particularly in mountainous regions where empirical evidence is still limited.

(Remarks on code availability)

Reviewer #3

(Remarks to the Author)

The authors have responded to most of my previous comments and the manuscript has improved in several respects. In particular, the discussion of different types of migration is clearer, and the paper now provides a more explicit explanation of whether migration occurs at the individual or household level. The expanded discussion on destinations and labour markets is also a welcome addition, as it helps situate migration decisions within broader economic contexts.
However, I remain unclear about how the concept of wellbeing is defined and operationalised in the paper. At present, the analysis appears to treat wellbeing primarily as a function of wealth or economic outcomes. If this is the intended conceptualisation, the authors should state this explicitly and justify the choice with reference to the relevant literature. Alternatively, if wellbeing is intended not to encompass broader dimensions apart from livelihoods and wealth (such as social, psychological… factors.) this should be clarified and reflected more clearly in the analysis and discussion.
I am not in a position to assess the econometric strategy employed in the paper and will defer to other reviewers who are better placed to evaluate the technical aspects of the methodology.
Figure 2 would benefit from additional explanation in both the main text and the figure caption. It is not entirely clear whether Figure 2 is intended to represent a hypothesis, a conceptual framework, and seems to be an illustration of the relationships discussed in the paper. Clarifying the purpose of the figure would help readers better understand how it should be interpreted. While I can follow the description provided in the text, the meaning of the purple arrow, labelled “substitution under shared resource constraint,” remains unclear in the visual representation. The authors should clarify how this relationship is conceptualised and how it should be interpreted within the framework presented in the figure. In addition, the origin and purpose of the dotted lines are not immediately evident are they based on data? It is important that readers can easily understand the relationships being illustrated.

(Remarks on code availability)

Version 2:

Reviewer comments:

Reviewer #1

(Remarks to the Author)
The authors have responded constructively to the previous comments and have improved the clarity of the empirical strategy, treatment of climate variables, and interpretation of the spatial model. The additional clarifications regarding non-causal interpretation and the nature of spatial correlation are appropriate and strengthen the manuscript. I have no further major concerns and consider the paper suitable for acceptance.

(Remarks on code availability)

Reviewer #3

(Remarks to the Author)
The revised text tackles the reviewers comments and is much improved.

Couple of minor comments:

I feel the order of the abstract is not quite there. Also the abstract the sentence that starts with "we find that extreme weather events, both locally and in neighboring districts is most strongly associated with international mobility and immobility"... " does not make sense.

Figure 2 is clearer

Table 1 require more explanation - either with better header, more explanations and a description of that the table is telling us overall.

(Remarks on code availability)

Open Access This Peer Review File is licensed under a Creative Commons Attribution 4.0 International License, which permits use, sharing, adaptation, distribution and reproduction in any medium or format, as long as you give appropriate credit to the original author(s) and the source, provide a link to the Creative Commons license, and indicate if changes were made.
In cases where reviewers are anonymous, credit should be given to 'Anonymous Referee' and the source.
The images or other third party material in this Peer Review File are included in the article’s Creative Commons license, unless indicated otherwise in a credit line to the material. If material is not included in the article’s Creative Commons

license and your intended use is not permitted by statutory regulation or exceeds the permitted use, you will need to obtain permission directly from the copyright holder.
To view a copy of this license, visit https://creativecommons.org/licenses/by/4.0/

Response to Reviewer 1

Overall Assessment

The paper presents interesting case of climate change and mobility nexus in less studies region. However, it requires substantial revision for robustness of empirical approach, and better identification strategy of causality given the local context. Moreover, contribution of the paper is not clear well, given the growing literature on climate-mobility relationship, including Central Asia and Kyrgyzstan. Presentation of attrition observations in the data over the 11 period (2013-2022) and better description of the country case are needed.

We thank the reviewer for this overall assessment and for highlighting important areas for improvement. In response, we substantially revised the manuscript to strengthen the robustness of the empirical approach, clarify identification and interpretation, sharpen the contribution relative to the existing climate–mobility literature, and improve transparency regarding the data and country context.

First, we expanded the robustness and diagnostic analyses by addressing spatial dependence, substitution patterns, climate-threshold choices, and the panel structure, and we explicitly assessed post-estimation spatial autocorrelation. Throughout the revised manuscript, we framed the results as conditional associations rather than causal estimates, given the observational nature of the data and the local context.

Second, we clarified the paper's contribution by positioning the analysis more explicitly within the climate–mobility literature. We emphasized how wealth-based mobility constraints interacted with spatially correlated climate shocks to produce heterogeneous mobility and immobility outcomes in a mountainous, infrastructure-constrained setting. This framing highlighted what the study added beyond well-established findings on migration networks and poverty-related immobility.

Third, we improved data transparency substantially. We documented the rotating-panel structure of the KIHS in detail, reported household retention rates across waves, and showed that the estimation sample was dominated by repeatedly observed households. We also clarified the use of survey weights and demonstrated sample stability across time, elevation bands, and well-being groups.

Finally, we expanded the description of the Kyrgyzstan context, including spatial variation in mobility, multidimensional well-being, elevation, and exposure to weather extremes, and we supported this discussion with new descriptive tables and figures in both the main text and the Supplementary Information.

We believe these revisions materially strengthened the credibility, interpretability, and contribution of the manuscript and directly addressed the reviewer’s concerns.

2. Significance and Novelty

The study’s focus on mountainous Central Asia—particularly Kyrgyzstan— provides interesting contribution to the climate–mobility literature. It uses integration of a multidimensional well-being index with a spatial multinomial logit model. The wealth-based mobility constraints framework provides an intuitive visualization of how wealth thresholds shape mobility behavior. However, it is important to clarify how the treatment of spatial interdependence in mobility decisions extends beyond established notions of “shared exposure” or “network diffusion.” The authors might elaborate on what is new or distinctive in their conceptualization of spatial effects—whether in mechanism, measurement, or interpretation.

We thank the reviewer for raising this important point. Our treatment of spatial interdependence was not intended to introduce a new behavioral mechanism, such as network diffusion or imitation in mobility decisions. Rather, the novelty of our approach lay in how spatial dependence was conceptualized, measured, and interpreted in a high-altitude, infrastructure-constrained setting.

Conceptually, we distinguished between two sources of spatial interdependence. Climatic spillovers captured the geographic co-occurrence and propagation of weather extremes across neighboring districts, reflecting shared exposure driven by physical processes. Socioeconomic spillovers, in contrast, captured correlated household conditions and constraints operating through accessibility, infrastructure, and migration-relevant networks. Treating these processes as distinct allowed us to move beyond a generic notion of “shared exposure” and to interpret spatial effects in terms of correlated constraints rather than behavioral diffusion.

Methodologically, we operationalized this distinction by using spatially lagged covariates rather than spatially lagged mobility outcomes and by constructing separate spatial weight matrices for climate and socioeconomic variables. Climatic spillovers were based on geographic proximity, whereas socioeconomic spillovers were based on travel-cost connectivity derived from a friction surface. This separation was particularly important in mountainous regions, where Euclidean distance poorly reflected effective connectedness and where climatic exposure and market access did not coincide spatially.

Finally, our interpretation of spatial effects differed from diffusion-based models. As shown by our robustness diagnostics, spatial correlation was strong prior to estimation and was

substantially attenuated after we accounted for spatially lagged covariates. We therefore interpreted spatial effects as reflecting shared environmental stress, correlated livelihood constraints, and regionally clustered opportunity sets, rather than imitation or contagion in mobility behavior. In this sense, the spatial structure in our model improved identification and interpretation of climate–mobility associations under correlated exposure, rather than positing a new form of behavioral spillover.

3. Theoretical Framework

Paper argues that the integration of the New Economics of Labour Migration (NELM) with the spatial poverty trap framework into a unified wealth-based mobility model as a contribution. Nonetheless, the framework could be positioned more clearly within the broader climate-mobility and adaptation literature. Explicitly linking this synthesis to other frameworks (e.g., capability approaches, adaptation pathways) would help readers situate the contribution conceptually.

We thank the reviewer for this helpful suggestion. Our intention was not to introduce a new standalone theoretical framework, but to integrate and operationalize existing perspectives in a way that was empirically tractable for climate-vulnerable, infrastructure-constrained settings.

In the revised manuscript, we positioned the resource-based mobility constraints framework more explicitly within the broader climate–mobility and adaptation literature. Conceptually, the framework aligned with capability-based approaches by treating mobility as conditional on households' ability to convert resources into feasible action under climate stress, while not directly modeling aspirations. By emphasizing wealth thresholds, constrained choices, and involuntary immobility, the framework also connected to adaptation research that highlighted limits to adaptation and differentiated adaptive capacity (lines 83–87).

Accordingly, we clarified that our contribution lay in synthesizing established ideas from the New Economics of Labour Migration and spatial poverty-trap models with capability-oriented perspectives, and in translating this synthesis into a unified empirical framework that allowed multiple mobility outcomes—including immobility—to be analyzed jointly under spatially correlated climate exposure. We revised the conceptual discussion to make these links explicit and to better situate the framework within the wider climate–mobility and adaptation literature (lines 305–325).

The conceptual figure is helpful but could be strengthened by including a concise formal representation of the model in the main text (in addition to the supplement). The substitution

mechanism between domestic and international mobility also merits clearer theoretical justification.

We thank the reviewer for this suggestion. We added a concise formal description of the wealth-based mobility constraints model to the main text to complement the conceptual figure, while retaining the full derivation in Supplementary Information S1.

The revised text clarified that domestic and international mobility drew on the same household resource pool but differed in fixed and variable costs. As a result, these outcomes were mutually exclusive at the individual level and substituted for one another as household resources increased. Domestic mobility became feasible at lower resource thresholds, whereas international mobility required substantially more resources. This mechanism followed directly from household budget constraints and cost differentials and did not rely on behavioral diffusion assumptions (lines 81-83).

4. Methods and Data Transparency

The methodological framework is robust and consistent with the study's aims, but several clarifications are needed to enhance reproducibility and interpretability.

Endogeneity and omitted variables. While the inclusion of lagged well-being indicators (MWI) helps address simultaneity to some extent, potential endogeneity between household well-being and mobility through mechanisms such as remittances, labor loss, or self-selection may still be present. It would be helpful for the authors to acknowledge this limitation and briefly discuss possible strategies to mitigate bias, and provide robustness checks. In addition, it is not clear to what extent the current model specification appears to omit several socio-economic and demographic factors that could plausibly influence both exposure to climate shocks and mobility behavior. (For instance, additional controls such as household head's age, education, number of children etc.).

We thank the reviewer for this important point. We explicitly acknowledged that, despite lagging household well-being (MWI), residual endogeneity between well-being and mobility could persist through channels such as remittances, labor loss, or self-selection into migration. For this reason, we interpreted all estimated effects as conditional associations rather than causal relationships, and we clarified this limitation in the revised manuscript (lines 536-549).

To mitigate simultaneity concerns, the main specification used lagged MWI to ensure that mobility outcomes were associated with pre-existing household conditions. In addition, we conducted several robustness checks, reported in the subsection *Robustness and Sensitivity* (lines 550-557). These included auxiliary regressions linking current well-being to future

mobility outcomes and re-estimation using alternative constructions of the MWI. The results consistently indicated that reverse causality was unlikely to be the primary driver of the observed associations.

Regarding omitted variables, the model included a broad set of household-level controls (household size, dependency ratio, gender composition, and rural residence), year effects, elevation interactions, and spatial spillovers capturing correlated socioeconomic and environmental conditions across districts (lines 509 - 516). Several demographic characteristics raised by the reviewer, such as education, were incorporated directly into the MWI by construction. Including these variables separately in sensitivity analyses did not materially alter the results and risked over-controlling for dimensions of well-being that were conceptually central to the framework. We clarified this rationale in the Methods section.

Climate thresholds and robustness. The selection of climate-shock thresholds (e.g., SPI < 1; STI > 2) would benefit from further clarification. It would be helpful if the authors could indicate whether these cut-offs were derived empirically, supported by prior studies, or based on theoretical reasoning. In addition, the rationale for using annual rather than seasonal aggregation could be explained more clearly, as could the choice to aggregate SPI values at the provincial rather than district level. Considering the potential overlap of extreme events such as concurrent drought and heat years as possible confounding factors would further strengthen the analysis. Finally, exploring robustness to alternative threshold values, aggregation levels, and seasonal definitions would help confirm that the results are not sensitive to the specific threshold selection.

We thank the reviewer for this detailed comment regarding the definition, aggregation, and interpretation of climate-shock thresholds.

In response, we revised the specification of extreme weather variables to reduce reliance on outcome-specific cut-offs (lines 449–456). In the revised analysis, extreme weather events were no longer modeled as purely binary indicators. Instead, we applied a two-step ("semi-binary") approach in which SPI and STI values within the normal range ($|SPI|, |STI| \leq 1$) were set to zero, while values exceeding this threshold were retained as continuous measures. This specification captured the intensity of climatic extremes conditional on exposure, rather than imposing discrete thresholds that could differ across hazards (lines 457–465).

The threshold of ±1 followed established conventions in the climate and hydrology literature, where SPI or STI values beyond ±1 are commonly interpreted as at least moderate anomalies[1]. This choice was therefore grounded in prior literature rather than derived empirically from the data. By contrast, the earlier use of different cut-offs across hazards

reflected conventional definitions used in previous studies but risked introducing unnecessary heterogeneity across weather variables. The revised semi-binary approach harmonized thresholding across climate indicators while preserving information on shock severity.

Aggregation choices reflected the underlying climatic processes relevant for Kyrgyzstan. Dry spells were aggregated annually to capture cumulative hydrological stress affecting agriculture and pasture systems, while excessive rainfall, cold winters, and hot summers were defined seasonally to reflect event-specific mechanisms. Dry-spell SPI was aggregated at the provincial level because droughts operated over broad hydrological basins, whereas other extremes were measured at the district level, where localized deviations more directly affected households[2].

We also addressed the reviewer's concern regarding potential confounding from concurrent weather extremes. Prior to estimation, we explicitly assessed overlap and statistical dependence among weather indicators. Although multiple extremes occasionally occurred in the same year, higher-order concurrence was rare. Pairwise correlations among climate variables were modest ($|\rho| \leq 0.20$), and variance inflation factors remained close to unity (VIF ≤ 1.09), indicating no multicollinearity concerns. These diagnostics confirmed that estimated associations were not driven by overlapping extreme events. We reported and discussed these results in the robustness section and Supplementary Tables S8–S9 (lines 599–606).

Model assumptions and diagnostics. Clarifying certain model assumptions would further strengthen confidence in the results. Though the inclusion of spatial terms helps to partially relax the Independence of Irrelevant Alternatives (IIA) constraint, the substitution patterns among mobility outcomes may still be imperfectly represented. Because domestic, international, and combined mobility are likely to be structurally related. Moreover, it is very important what is mean by the "combined" migration. Because if it includes international and, after, internal, then it may have different implications. As return migrant may have some other unobserved heterogeneity that may have implications for estimation results.

We thank the reviewer for this important clarification. In the revised manuscript, combined mobility referred specifically to households reporting both domestic and international moves by different household members within the same observation period. It did not capture sequential mobility, return migration, or cases in which the same individual first migrated internationally and subsequently relocated domestically. We explicitly clarified this definition in the Methods section to avoid ambiguity and to distinguish combined mobility from return or stepwise migration processes (lines 430–434).

Regarding model assumptions, we agreed that domestic, international, and combined mobility outcomes were structurally related rather than independent choices. While the inclusion of spatially lagged covariates allowed substitution patterns to vary systematically with local and neighboring conditions—thereby partially relaxing the Independence of Irrelevant Alternatives (IIA) assumption—we did not assume that IIA held mechanically.

To assess sensitivity to this assumption, we conducted drop-one-alternative diagnostics in which each mobility outcome (except the reference category) was sequentially excluded and the model re-estimated (lines 582–589). As expected, we observed larger coefficient changes when closely related alternatives (e.g., domestic versus international mobility) were removed, reflecting partial violations of IIA. This behavior was consistent with interpreting migration choices as related behavioral margins rather than independent alternatives.

Importantly, despite these structural relationships, the direction, relative magnitude, and qualitative interpretation of the key associations between extreme weather exposure, household resources, and mobility outcomes remained stable across all specifications. Accordingly, the analysis emphasized robust patterns of mobility and immobility under climate stress rather than precise substitution elasticities between closely related mobility categories.

It would also be helpful to explain the rationale behind selecting K = 8 in the KNN robustness check and to present diagnostic statistics for spatial dependence quantitatively rather than descriptively. In addition, clarification of several cutoff choices - such as the 2,000 m elevation threshold, the use of household-head education only, and the benchmark of 11 years of schooling - would improve transparency. Finally, reporting post-estimation diagnostics such as Moran’s I on residuals or generalized residuals would provide useful evidence that spatial autocorrelation has been adequately mitigated.

We thank the reviewer for these suggestions and have revised the manuscript to improve transparency and diagnostic reporting.

The rationale for choosing K = 5 nearest neighbors was to capturing local spatial dependence while avoiding overly large neighborhoods that may dilute localized climatic and socioeconomic interactions, particularly in sparsely populated high-altitude regions. Robustness checks using alternative neighborhood sizes (K = 4–10) yielded qualitatively very similar results, indicating that findings are not sensitive to the specific choice of K (Supplementary Tab. S5).

We now report quantitative diagnostics for spatial dependence. Pre-estimation Moran’s I statistics computed for district-level mobility outcomes and climate variables reveal strong and significant spatial autocorrelation. Post-estimation Moran’s I statistics based on

generalized residuals show that residual spatial dependence substantially reduced after accounting for spatial spillovers, suggesting that remaining (Supplementary Tab. S5 – S6).

Finally, the 2,000 m elevation threshold follows established conventions in mountain research and captures sharp changes in climate, accessibility, and livelihoods in Kyrgyzstan. To avoid reliance on a single cutoff, we introduce nested elevation bands (above 1,000 m, 1,500 m, and 2,000 m a.s.l.). Household-head education is used to capture long-term human capital relevant for household mobility decisions, and the eleven-year schooling benchmark reflects Kyrgyzstan’s compulsory education system and is embedded in the construction of the MWI (lines 435-448).

Panel design and attrition. The study uses n = 45,161 household-year observations from the Kyrgyz Integrated Household Survey (KIHS, 2013–2022), covering about 5,000 households annually within a rotating-panel structure. Because the KIHS replaces a portion of households each year, attrition and sample rotation may affect representativeness. Evidence from earlier KIHS documentation (e.g. https://www.econstor.eu/bitstream/10419/129271/1/diw_datadoc_2011-062.pdf) suggests that annual retention rates range from roughly 60 % to 90 %, with about 25 % replacement. Based on this, it is important to demonstrate that sample size used in the study consists of households that are constantly observed in the survey since 2013 up to 2022.

The authors should clarify how these dynamics were treated—whether through weighting adjustments, re-weighting for representativeness, or robustness checks—and consider including a summary table showing the number of repeated and new households by year and across MWI terciles to demonstrate sample stability.

Additionally, more description of the data is needed if the KIHS were not designed for the communities at the high altitude focusing on the specific area of the region.

We thank the reviewer for these points regarding panel design, attrition, and representativeness. The KIHS followed a rotating-panel design, and households were therefore not expected to be observed continuously from 2013 to 2022. Our analysis did not rely on a balanced panel or on households being present throughout the entire period; instead, it exploited both within-household variation across observed waves and between-household variation across space and time, which was appropriate for our empirical strategy (lines 399–406).

To address concerns about attrition and sample stability, we documented the panel structure explicitly. Supplementary Table S1 reported pairwise household retention rates across survey waves, showing high retention between adjacent years and a gradual decline over longer

intervals consistent with planned survey rotation rather than selective attrition. The estimation sample was dominated by repeatedly observed households: the median household was observed in eight waves, the mean participation length exceeded six years, and fewer than 1% of households exhibited non-consecutive gaps.

All estimations used the official KIHS survey weights, preserving national representativeness under the rotating-panel design. No additional re-weighting was applied, as household entry and exit were determined by survey design rather than selective dropout. Descriptive statistics by elevation band and MWI tercile showed stable distributions over time, indicating that sample rotation did not drive the results (Supplementary Table SS1).

Finally, we expanded the data description in the Methods section to clarify coverage of high-altitude areas. While the KIHS did not oversample mountain communities, its nationally representative sampling framework ensured coverage of mountainous and remote districts across all provinces, allowing us to examine elevation-related heterogeneity while acknowledging that the survey was not specialized for any single subregion (lines 389–390).

Spatial specification. The construction of the spatial weights matrix (K = 10) should be described in greater detail. For example, looking in Figure 3 and satellite image/map, districts located on opposite banks of Issyk-Kul Lake (Cholpon-Ata city and Darhan village), or those in Ak-Talaa (Naryn), Uzgen (Osh), and Suzak (Jalal-Abad), as well as Talas and Zhumgal raions, appear geographically distant and separated by major natural barriers or may have different conditions for social and economic livelihood. It would be helpful to clarify whether the KNN matrix is based purely on centroid-to-centroid Euclidean distances or whether adjustments were made for topographic features and administrative boundaries.

We thank the reviewer for this important clarification. In the revised manuscript, we expand the description of the spatial weights matrices and explicitly address the role of geographic barriers and accessibility.

Spatial dependence is modeled through spatially lagged covariates, and we distinguish between socioeconomic and climatic spillovers. For socioeconomic variables (including MWI), we constructed spatial lags using a K-nearest-neighbors matrix based on travel costs, not simple centroid-to-centroid Euclidean distance. Travel costs were derived from a global friction surface that incorporates road networks, slope, land cover, and transport infrastructure. As a result, districts separated by major natural barriers, such as mountain ranges or Issyk-Kul Lake, were not treated as closely connected when accessibility is limited, even if their geographic distance is small.

For climate variables, we constructed spatial lags using a separate distance-based KNN matrix with inverse-distance weights, reflecting the physical propagation of meteorological processes rather than socioeconomic interaction. This distinction ensures that spatial relationships correspond to the underlying mechanisms of interest—accessibility and networks for socioeconomic spillovers, and geographic proximity for climatic spillovers. These clarifications have been added to the Methods section to improve transparency and interpretability (lines 486-501).

Administrative definitions. Finally, the description of administrative units should be made more precise. It remains unclear whether the term district refers to the KIHS primary sampling unit (aiyl aimak or city) or to the administrative unit level. Similarly, province should be explicitly defined as corresponding to the first or second level of administrative unit. Since spatial dependence (KNN = 10) and the aggregation of climate indices depend on the geographic scale, clarifying these definitions would greatly enhance interpretability and reproducibility.

We thank the reviewer for highlighting the need for clearer administrative definitions. In the revised manuscript, we now specify the geographic units used in the analysis more precisely.

In this study, the term district refers to the aiyl aymak (or city) level, which constitutes the lowest administrative unit with consistent geographic identifiers in the KIHS and serves as the spatial unit of analysis. These units also correspond to the primary sampling units (PSUs) of the survey. Aiyl aymaks and cities are nested within provinces (oblasts), which represent the first-level administrative division of Kyrgyzstan.

District-level (aiyl aymak) identifiers are available for all household–year observations in the estimation sample (2013–2022), allowing linkage to 205 spatial units and to the corresponding spatial covariates. All spatial weights matrices (KNN) and spatially lagged variables are therefore constructed at the aiyl aymak level.

Climate indices were aggregated at different spatial scales depending on the underlying climatic process. Dry spells were aggregated at the provincial (oblast) level to capture basin-wide hydrological stress, while excessive rains, cold winters, and hot summers were measured at the district (aiyl aymak) level, where localized exposure is most relevant. These choices and their rationale are now explicitly described in the Methods section.

5. Results and Interpretation

As noted above empirical results should be controlled for endogeneity and selection bias issues and sensitivity and robustness analysis are very important.

One of the potential concerns in interpreting results is that neighboring effect of extreme weather events may have effect on mobility. Generally, as literature and current analysis suggest mobility in Central Asia mostly appear as international labor force mobility. From this standpoint, mobility in Kyrgyzstan is mostly represented by international migration, where Russia is the main destination. On the other hand, internal mobility concentrated towards the Chui valley central region with capital city. If we agree on this, then I am a bit skeptical about the importance of spatial effect of extreme climate on mobility of those living in high altitudes (say those living in more than 2000 meters). As locations with strong migration network may facilitate mobility of people even (which is the commonly argued consensus in the migration literature), therefore whatever the extreme weather happens in neighboring district or community, people may use migration or mobility options; also, immobility of those with low income (or wealth) due to financial constraint is not novel in the literature. Moreover, those living in high altitudes, may have already preferences living in those area with the decision to have livelihood based on animal husbandry, which might be decision for medium or long-term period, and short-term weather extremes may have less effect?

We thank the reviewer for this thoughtful comment. We agree that international labor migration—primarily to Russia—dominates mobility in Kyrgyzstan and that migration networks play a central role in facilitating such movements. Our analysis does not challenge this well-established pattern, nor does it suggest that short-term weather shocks override long-term livelihood strategies or migration networks.

The spatial effects we identify instead capture how spatially correlated extreme weather exposure across neighboring districts alters the local opportunity set under constrained conditions, particularly in high-altitude regions. Even where international migration networks exist, widespread climatic stress in surrounding areas can reduce liquidity, weaken informal risk-sharing mechanisms, and intensify local competition for coping strategies. These processes limit households' ability to activate mobility options, especially among poorer households, rather than shaping destination choice or network formation.

Importantly, our results are heterogeneous. Wealthier households often continue to realize mobility despite local or regional shocks, consistent with the facilitating role of migration networks. By contrast, poorer households remain immobile when extreme weather affects their neighborhood, even when they have aspirations to migrate. This conditional pattern, where the same spatially clustered shocks enable mobility for some households while constraining it for others, is central to our contribution.

Finally, while the association between poverty and immobility is well documented, our contribution lies in showing how wealth thresholds interact with spatially correlated climate exposure to generate divergent mobility outcomes within a highly mountainous setting. We therefore interpret our findings as refining existing migration theories by clarifying when dominant migration pathways fail to translate into realized mobility under compounded spatial stress.

6. Presentation and Style

The manuscript reads well, with clear and coherent flow. Minor stylistic improvements could enhance readability.

We thank the reviewer for this positive assessment. In the revised manuscript, we carefully reviewed the text for clarity, consistency, and flow, and made minor stylistic revisions where appropriate to improve readability without altering the substantive content.

It would also be valuable to include in the Supplementary Materials detailed tables of marginal effects and confidence intervals corresponding to all mobility outcomes in Figure 4, consistent with best practices in the economics and environmental-migration literature.

We thank the reviewer for this helpful suggestion. In the revised manuscript, we now included detailed tables reporting marginal effects and corresponding confidence intervals for all mobility outcomes presented in Figure 4. These results are provided in the Supplementary Source Tables (Tab_SS2 and Tab. SS3).

The authors note (lines 87-88) that nearly 10% of the population lives above 2,000 m a.s.l., where mobility constraints are most pronounced. To strengthen this statement, it would be useful to include a summary of the sample distribution by altitude levels, and disaggregated by migration outcome, exposure to weather shocks, and well-being (MWI) categories. Presenting these descriptive statistics - either in the main text or the Supplementary Materials - would align with standard practice in the economics and development literature, where summary tables are typically provided to illustrate sample representativeness and variable heterogeneity. Such inclusion would add clarity and enhance the transparency of the empirical analysis.

We thank the reviewer for this helpful suggestion. In the revised manuscript, we now report descriptive statistics by elevation band in the main text (Tab. 1), documenting differences in mobility outcomes, household well-being index (MWI), household characteristics, and exposure to extreme weather across altitude levels.

The table shows that households living above 2,000 m a.s.l. exhibit the highest immobility rates, the lowest average MWI, and the largest concentration of low-MWI

households, supporting the statement that mobility constraints are most pronounced at higher elevations. International mobility peaks at mid-elevations (1,000–1,500 m), while combined mobility remains rare across all altitude bands.

To further enhance transparency, Supplementary Source Table SS1 provides detailed descriptive statistics disaggregated by mobility outcome and MWI group, including elevation, household characteristics, and exposure to weather shocks. Together, these additions illustrate sample heterogeneity and align the presentation with standard practice in the economics and development literature.

7. Discussion and Policy Implications

The discussion is articulate and policy-relevant but could more explicitly connect findings to regional adaptation frameworks (e.g., UNDP, GCF, or national adaptation plans) and to the broader literature on "trapped populations" and immobility under climate change.

We thank the reviewer for this suggestion. In the revised manuscript, we strengthened the discussion and conclusion to more explicitly situate our findings within both policy frameworks and the literature on immobility and 'trapped populations.' Specifically, we now link our results on constrained and involuntary immobility to growing policy attention to trapped populations in climate adaptation planning, drawing on the World Bank's *Groundswell* framework (Rigaud et al., 2018) and related work emphasizing safe immobility as a legitimate adaptation outcome.

We also connected our findings to National Adaptation Plan (NAP) processes supported by UNDP and the Green Climate Fund, highlighting how human development, social protection, and in-situ support feature prominently in contemporary adaptation strategies. These additions clarify how the wealth-based mobility constraints framework complements existing climate–adaptation approaches by identifying when mobility is feasible, when immobility is likely, and where targeted support for safe immobility is most needed.

Overall Recommendation:

Overall, the paper has potential for publication. However, it requires substantial revision for robustness of empirical approach, and better identification strategy of causality given the local context.

We thank the reviewer for the meticulous assessment of our manuscript and for recognizing the paper's potential contribution. In response to the concerns regarding robustness and identification, we substantially revised the manuscript to strengthen the empirical approach and improve transparency. We believe these revisions have substantially improved its scientific contribution.

Specifically, we expanded robustness and diagnostic analyses addressing spatial dependence, substitution patterns, climate-shock thresholds, panel structure, and sample stability; clarified model assumptions and limitations related to endogeneity and selection; and added extensive descriptive statistics and post-estimation diagnostics in both the main text and the Supplementary Materials. We also clarified the interpretation of the results throughout the manuscript, explicitly framing them as conditional associations rather than causal estimates, consistent with the data structure and local context.

We hope these changes adequately address the reviewer's concerns and strengthen the manuscript's contribution.

Minor comments:

In Figure 1, the continuity and termination of curves should be clarified. Specifically, it is unclear why the dashed blue line (domestic mobility under shocks) stops abruptly at point D while the solid blue line continues beyond F, and why both solid blue and green lines terminate near thresholds e and h. If these endpoints are meant to represent upper bounds of household wealth or limits beyond which mobility probabilities stabilize, this should be explicitly stated. Otherwise, extending the curves or adding a short explanatory note in the caption would help indicate whether mobility probabilities stabilize, decline, or remain constant beyond these thresholds. These clarifications would improve the figure's internal consistency and help readers better grasp the wealth-mobility relationship being illustrated.

We thank the reviewer for this helpful comment. In response, we revised the conceptual figure and its caption to clarify the continuity and termination of the mobility curves. The updated caption now explicitly states that the curves are illustrative and shown over a relevant range of household wealth, and that their endpoints do not represent hard bounds on wealth or mobility probabilities. The revised figure avoids abrupt or ambiguous terminations, improving internal consistency and making clear that mobility probabilities are not assumed to stabilize or decline beyond the illustrated thresholds. These changes enhance the interpretability of the wealth–mobility relationship depicted in the figure.

Figure 4 is compelling but would benefit from minor design adjustments. The main and spatial-lag effects (MWI through Rural-SL) are tightly clustered around zero, making small but meaningful differences difficult to discern. The elevation-interaction terms (e.g., MWI × Elev, CW × Elev) are already clear. Readability could be improved by narrowing the x-axis range (e.g., -5% to 5%), adding an inset zoom, or splitting the figure into two panels with separate scales (main/spatial-lag and elevation interactions).

We thank the reviewer for this helpful suggestion. In response, we fully redesigned Figure 4 to improve clarity and interpretability. The revised figure replaces the original layout and presents effects on scales that allow small but meaningful main and spatial-lag associations to be clearly distinguished, while preserving clear visualization of elevation-interaction terms. This redesign substantially improves readability and comparison across mobility outcomes without altering the underlying estimates or their interpretation.

The authors note (lines 174-176) that cold winters at high elevation modestly increase domestic mobility among rich households by +4 %, but in Figure 4 the effect appears closer to -4 %. This discrepancy should be clarified. It would also help to specify in lines 184-193 that these findings refer to high-altitude interaction effects.

We thank the reviewer for noting this inconsistency. The discrepancy arose from an earlier draft of the results text that was not fully aligned with the final specification underlying Figure 4. In the revised manuscript, the results text has been corrected to reflect the estimates shown in the figure, and the previously stated positive association between cold winters at high elevation and domestic mobility among rich households has been removed.

We also revised the relevant paragraphs (lines 184–193) to state explicitly when reported associations refer to elevation–climate interaction effects, ensuring consistency between the text, figures, and interpretation.

Figures 3 and 4 are informative but could be reorganized to improve clarity. Figure 3 might be divided into three figures (1) mobility outcomes, (2) multidimensional well-being, and (3) weather extremes.

We thank the reviewer for this helpful suggestion. In the revised manuscript, we improved the organization and readability of Figure 3 by introducing clearer visual separation between panel groups, enhanced labeling, and more explicit panel headings. This preserves all information within a single integrated figure—consistent with common practice in *Nature* journals—while making the distinctions between mobility outcomes, multidimensional well-being, and weather extremes more transparent and easier to follow.

In Figure 3e, please clarify whether the rise in the education component of the MWI reflects an increase in the weight of education or an actual rise in the underlying sub-index.

We thank the reviewer for this clarification. In the revised manuscript, we explicitly state that the observed rise in the education component of the MWI reflects changes in the underlying education sub-index rather than changes in indicator weights. All component weights were fixed by construction throughout the analysis, and temporal variation in Figure

3e therefore arises solely from changes in the underlying sub-indices. This clarification has been added to the Results text accompanying Figure 3 (Fig. 3d–f).

1 McKee, T. B., Doesken, N. J. & Kleist, J. in *Proceedings of the 8th Conference on Applied Climatology*. 179-183 (California).

2 Svoboda, M., Hayes, M. & Wood, D. Standardized precipitation index: user guide. (2012).

Response to Reviewer 2

The paper is well-written, ambitious, and presents a novel wealth-based framework for understanding mobility under extreme weather in Kyrgyzstan. It combines household survey data with high-resolution climate information and a spatial econometric model, which is a strong methodological choice. However, several limitations undermine the strength of its claims. The paper tends to overstate causal inference, underexplains methodological decisions, and overlooks some crucial conceptual dimensions (e.g., social networks, governance, migration policies) that are essential to understanding mobility.

We thank the reviewer for the positive assessment of the paper's ambition, conceptual framework, and methodological approach. We also appreciate the careful identification of limitations that required clarification and greater restraint. In response, we substantially revised the manuscript to address these concerns.

First, we revised the language throughout the paper to avoid overstating causal inference. Given the observational nature of the data and the institutional and geographic context, we consistently framed the results as conditional associations rather than causal effects, and we explicitly discussed remaining endogeneity and selection concerns in the Methods section (Methods section, lines 541–557).

Second, we expanded and clarified the rationale for key methodological choices, including the construction of spatial weights, the selection of climate thresholds, elevation cutoffs, treatment of the rotating panel, and post-estimation diagnostics. These decisions and their implications were documented more transparently in the Methods section and Supplementary Information.

Third, we agreed that social networks, governance structures, and migration policies were important determinants of mobility. Given data limitations, we did not model these dimensions explicitly. Instead, we clarified in the revised Discussion how such factors were partially reflected through spatial spillovers, household resources, and regional heterogeneity, and we acknowledged them as important directions for future research.

We believe these revisions strengthened the manuscript's credibility, transparency, and conceptual positioning while preserving its core empirical contribution.

This paper is innovative and empirically rich, but it currently risks overclaiming and oversimplifying. To achieve publication-level strength, it requires a more balanced conceptual framework, cautious causal language, stronger contextualization, and greater policy relevance. I hope the following remarks are helpful:

I was wondering if the framework is highly wealth-centric, with mobility outcomes explained almost exclusively through the household Multidimensional Well-being Index (MWI). While this is valuable, it risks reductionism. As there is now considerable evidence in the migration literature that suggests social networks, governance regimes, migration policies, and aspirations play a critical role, alongside wealth. It is expected to broaden the conceptual framing beyond wealth thresholds to include migration aspirations, social capital, cultural attachments, and institutional enablers/barriers. This would prevent over-simplification, perhaps by providing a conceptual diagram that integrates wealth with non-economic drivers of mobility (e.g., network effects, governance quality, migration regimes).

We thank the reviewer for this important and thoughtful comment. We agreed that mobility decisions were shaped by a broader set of factors than household resources alone, including social networks, governance regimes, migration policies, cultural attachments, and aspirations. In the revised manuscript, we therefore clarified the conceptual scope of the framework to avoid a wealth-centric or reductionist interpretation (Framework and Discussion section).

Specifically, we situated the analysis more explicitly within the aspirations–capabilities approach, drawing on the New Economics of Labour Migration and spatial poverty-trap perspectives. Within this framing, mobility outcomes reflected the interaction between aspirations to move and capabilities to realize mobility. Aspirations were not assumed away, but were treated as largely latent and potentially widespread under climatic stress, while heterogeneous capabilities—shaped by material resources, health, access to services, and the ability to convert resources into effective action—determined whether and which mobility outcomes were realized (Framework).

Accordingly, the empirical analysis focused deliberately on variation in mobility capabilities rather than claiming to provide a comprehensive explanation of migration behavior. The Multidimensional Well-being Index (MWI) was used as an operational proxy for household resources relative to mobility costs, not as a complete measure of all drivers of mobility. We made this distinction explicit in the framework and discussion sections.

We also clarified that non-economic drivers emphasized in the migration literature—such as social capital, migration networks, governance quality, and institutional constraints—operated alongside household resources and could shape both aspirations and constraints. While these factors could not be directly observed in the available nationally representative

data, they were discussed conceptually and acknowledged as important complements to the resource-based constraints identified here, as well as key directions for future research.

Finally, to further reduce the risk of over-simplification, we revised the conceptual figure and accompanying text to emphasize that resource thresholds operated within a broader social, institutional, and spatial context rather than as standalone determinants of mobility.

It implies the causal relationships between extreme weather events and mobility responses, yet the methodology (spatial multinomial logit with lagged predictors) is correlational. But it cannot isolate causal mechanisms. So it is expected that the claims are associations rather than causal effects. If causal language is retained, justify it with robustness checks (such as instrumental variables, quasi-experiments, or longitudinal event-history models), and explicitly discuss concerns about endogeneity (e.g., mobility could affect well-being, not only the reverse).

We thank the reviewer for this important clarification and fully agree that the empirical strategy did not identify causal effects in a strict sense. In the revised manuscript, we therefore revised the language throughout to avoid causal claims and consistently framed the results as conditional associations between extreme weather exposure, household well-being, and mobility outcomes (Methods and Results sections).

We explicitly stated that the spatial multinomial logit model with lagged predictors could not isolate causal mechanisms and that endogeneity could persist through channels such as remittances, labor loss, or selective mobility affecting subsequent household well-being. These limitations were discussed directly in the Methods section and revisited in the Discussion.

To mitigate simultaneity concerns, we relied on lagged measures of household well-being, extensive household-, spatial-, and temporal controls, and a comprehensive set of robustness and diagnostic checks, including alternative specifications, climate-threshold definitions, spatial-dependence diagnostics, and sensitivity analyses. Importantly, we did not interpret these checks as establishing causality and refrained from causal language accordingly (lines 541–557).

Given data and institutional constraints, more demanding identification strategies—such as instrumental variables, quasi-experimental designs, or event-history models—are not feasible in this setting. We therefore position the contribution as documenting systematic patterns, heterogeneity, and spatial structure in mobility outcomes under climate stress, rather than estimating causal treatment effects. We believe this clarification aligns the interpretation of the results appropriately with the methodology and strengthens the paper’s transparency and credibility.

The Multidimensional Well-being Index (MWI) is adapted, but choices such as replacing child mortality with dietary diversity are unjustified. Mobility data rely on annual household reports and exclude short-term, circular, or undocumented migration, which may bias results. Additionally, climate extremes are defined with strict cutoffs (SPI, STI), which may overlook localized hazards and perceptions. It requires sensitivity analyses showing how results change under alternative definitions of mobility and well-being, as well as how unobserved mobility (temporary or undocumented) could impact conclusions.

We thank the reviewer for raising these important measurement concerns. In the revised manuscript Methods section, we clarified and justified the construction of the MWI, explicitly acknowledged limitations in the mobility data, and explained how these measurement choices affected the interpretation of results.

The adaptation of the MPI framework reflected data availability and contextual relevance in Kyrgyzstan. Child mortality exhibited very low incidence and limited variation in the KIHS and therefore provided little discriminatory power for differentiating household well-being. We therefore replaced this indicator with dietary diversity, measured using a Berry Index based on food expenditure shares, which captured meaningful variation in nutritional well-being and was commonly used in middle-income and post-Soviet contexts. We clarified this rationale in the Methods section and documented index construction and weighting in the Supplementary Tab. S2.

Regarding mobility measurement, the KIHS recorded annual household-reported residential moves and did not capture short-term, circular, or undocumented mobility. We explicitly acknowledged this limitation in the Methods section. While unobserved mobility could affect absolute mobility levels, our analysis focused on relative differences across elevation, household resources, and climate exposure. These gradients were unlikely to be driven by short-term or unreported movements, which tended to be less spatially and socioeconomically differentiated. Accordingly, the results were interpreted as reflecting longer-term or economically consequential mobility decisions rather than all forms of movement (lines 424–434).

With respect to climate extremes, we revised the specification to reduce reliance on hazard-specific cutoffs. Rather than binary indicators, we adopted a semi-continuous approach in which SPI and STI values within the normal range ($|SPI|, |STI| \leq 1$) were set to zero, while deviations beyond this threshold entered the model as continuous measures. This specification captured intensity conditional on exposure and avoided imposing discrete thresholds that could obscure heterogeneity across hazards (lines 457–465).

The ±1 threshold follows established conventions in the climate and hydrology literature, where SPI and STI values beyond this range are commonly interpreted as at least moderate anomalies1. This harmonized approach replaces earlier hazard-specific cutoffs and improves comparability across climate indicators.

Finally, some sensitivity analyses requested—such as alternative definitions of undocumented or temporary mobility—were not empirically feasible with the available survey data. We therefore framed the conclusions explicitly as conditional associations and emphasized that unobserved forms of mobility could affect levels but were unlikely to overturn the main patterns of heterogeneity across household resources, elevation, and spatial exposure documented in the study. We highlighted these limitations as priorities for future research using complementary data sources in Discussion section.

While the paper briefly references the Andes, Himalayas, and East Africa, this is superficial. It misses an opportunity to demonstrate the model's global applicability. It is possible to expand the comparative analysis—what lessons from Kyrgyzstan are generalizable to other mountainous regions, and what is unique to this case?

We thank the reviewer for this important suggestion. In the revised manuscript, we expanded the discussion to situate the Kyrgyzstan findings more clearly within the broader literature on mobility in mountainous regions worldwide (lines 326–341).

Rather than brief references, we explicitly discussed structural features common across mountain systems—such as high relocation costs, remoteness from economic centers, limited infrastructure, and recurrent exposure to climate extremes—and showed how these conditions generated comparable patterns of selective mobility and immobility in regions including the Andes, the Himalayas, and East Africa.

At the same time, we clarified what was distinctive about the Kyrgyzstan case, particularly the coexistence of widespread international labor migration with persistent immobility in high-altitude communities. This combination reflected a specific institutional and migration regime layered onto geographic and socioeconomic constraints shared with other mountain regions. The revised discussion therefore distinguished between mechanisms that were broadly generalizable (resource thresholds, cost-differentiated mobility, spatially correlated exposure) and those that were context-specific, strengthening the model's relevance beyond the case study without overstating external validity.

The recommendations (infrastructure, social protection, relocation assistance) are broad and could apply to almost any migration-climate paper. They don't directly emerge from the results. It is suggested to translate the findings into specific policy insights for Kyrgyzstan. For

example, what role should Kyrgyz migration agreements with Russia or Kazakhstan play? How can social protection programs be tailored to mountainous regions? Or any other context-specific adaptation and migration policy suggestions that align with Kyrgyzstan's governance, institutions, and labor market realities.

We thank the reviewer for this important comment and agree that policy implications should be clearly anchored in the empirical results and in Kyrgyzstan's specific institutional and migration context. In the revised manuscript, we sharpen the policy discussion to derive context-specific insights that follow directly from the observed patterns, rather than offering generic recommendations.

First, our findings show that international mobility declines during spatially widespread extreme weather events, particularly among households in high-altitude districts, even in a country where international labor migration is otherwise well established. This result has direct implications for Kyrgyzstan's bilateral labor migration arrangements with Russia and Kazakhstan. It suggests that during years of widespread climatic stress, international migration cannot be assumed to function as an automatic coping mechanism. Policy coordination with destination countries—such as maintaining flexible entry conditions, safeguarding seasonal work permits, or ensuring continuity of remittance channels during crisis periods—becomes particularly important for preventing involuntary immobility among vulnerable households.

Second, the strong concentration of immobility among low-MWI households at higher elevations implies that social protection systems in Kyrgyzstan need to be spatially and seasonally targeted, rather than uniform. Because mobility constraints are most pronounced in remote mountain districts, our results point to the importance of scaling up location-specific in-situ support—such as cash transfers, health outreach, and emergency assistance—during years with concurrent extreme events across neighboring districts. This recommendation follows directly from the finding that spillover exposure reinforces immobility even when households face similar shocks elsewhere.

Third, the finding that better-off households retain more diversified mobility options underscores the role of human development as an adaptation instrument. In Kyrgyzstan's governance context, where social assistance and basic service provision are largely centralized but implemented locally, investments in health access, education, and living standards in mountain regions can expand feasible adaptation strategies over time, including both voluntary mobility and safe immobility. This aligns with existing national development and adaptation priorities rather than proposing new institutional structures.

Finally, the spatial spillover effects documented in the analysis imply that district-by-district adaptation planning may be insufficient in mountainous regions where climatic exposure and constraints are spatially correlated. For Kyrgyzstan, this suggests that coordination across neighboring rayons and oblasts—particularly in high-altitude areas—may improve the effectiveness of disaster preparedness and response, even within existing administrative frameworks.

We have revised the Conclusion and Policy Implications section accordingly to make these links explicit. The revised discussion emphasizes how the results inform migration governance, spatially targeted social protection, and coordinated adaptation planning in Kyrgyzstan, rather than offering generic policy prescriptions. We believe this revision directly addresses the reviewer's concern and strengthens the policy relevance of the findings.

Although the authors acknowledge but do not adequately analyze cultural ties, place attachment, and voluntary immobility. These are critical in mountain communities, where a strong attachment to ancestral land is prevalent. A subsection on voluntary immobility and its complications for the wealth-based threshold model would be beneficial for readers, which could be achieved by integrating qualitative evidence (from other studies, secondary data, or literature) on how immobility is not only poverty-driven but also culturally/psychologically rooted.

We thank the reviewer for this important observation. We agree that cultural ties, place attachment, and voluntary immobility are central to understanding mobility outcomes in mountain regions. These mechanisms are explicitly discussed in both the Introduction and the Discussion, where we emphasize that immobility reflects not only economic constraints but also identity-based ties to land, livelihood specialization, social obligations, and long-term settlement strategies documented in qualitative and mixed-methods research[2-4].

Our empirical framework is intentionally designed to capture the material feasibility of mobility rather than to model aspirations or cultural preferences directly, which are not observable in the KIHS data. Accordingly, the wealth-based mobility framework should be interpreted as identifying whether households are able to move, not whether they wish to do so. We clarify in the Discussion that persistent immobility among households with moderate or high well-being—especially at high elevations—is consistent with voluntary immobility driven by place attachment and cultural factors, rather than contradicting the threshold-based logic of the model.

Given the quantitative and nationally representative nature of the data, integrating qualitative evidence or estimating voluntary immobility as a distinct behavioral category is

beyond the scope of the current analysis. Instead, we explicitly position voluntary immobility as complementary to, rather than competing with, the wealth-based framework: cultural and psychological factors shape mobility choices within the set of feasible options defined by household capabilities and geographic constraints. We believe this clarification adequately addresses the reviewer's concern while preserving the empirical focus and scope of the study.

Minor Issues

• Writing sometimes overstates novelty: distinguishing four outcomes (immobile, domestic, international, combined) is useful, but not entirely new in migration studies.

We thank the reviewer for this helpful clarification. We agree that distinguishing between immobility, domestic, international, and combined mobility outcomes is not novel per se in the migration literature. In the revised manuscript, we therefore revised the language to avoid overstating novelty and to clarify that our contribution lies in analyzing these outcomes jointly within a unified, wealth-based and spatially explicit framework under climate stress, rather than in the classification itself.

• Figures (especially Fig. 1 & 5) need more clarity—threshold dynamics could be misread without stronger explanations.

We thank the reviewer for this helpful comment. In response, we revised Figures 1 and 5 and their accompanying captions to more clearly explain the threshold and substitution dynamics illustrated in the analysis.

In the revised figures, mobility outcomes are presented with clearer labeling and structure, and we explicitly clarify that the depicted thresholds and curves are illustrative rather than implying sharp cutoffs or causal transitions. Additional explanatory text in the captions guides readers through how mobility outcomes evolve across wealth levels and elevation bands, reducing the risk of misinterpretation.

• The robustness section is buried in methods; it should be summarized in results.

We thank the reviewer for this suggestion and agree that robustness checks are important for interpreting the empirical results. At the same time, the robustness and diagnostic analyses in this study were extensive and technically detailed, covering spatial dependence diagnostics, alternative climate thresholds, neighborhood definitions, panel structure, substitution patterns, and endogeneity concerns.

Rather than reproducing or summarizing these analyses in the Results section—which would have required substantial repetition and detracted from the presentation of the core empirical findings—we retained the full robustness and sensitivity analyses in the Methods section and Supplementary Materials, where they could be presented with the necessary

technical detail (Methods section, lines XXX–XXX; Supplementary Information, lines XXX–XXX).

This approach followed common practice in the economics and environmental-migration literature, where Results sections focus on substantive patterns, while robustness and diagnostic evidence is documented in the Methods or Supplementary sections. We believe this struck an appropriate balance between transparency and readability, given space constraints and the technical nature of the checks.

1 McKee, T. B., Doesken, N. J. & Kleist, J. in *Proceedings of the 8th Conference on Applied Climatology.* 179-183 (California).

2 Blondin, S. Staying despite disaster risks: Place attachment, voluntary immobility and adaptation in Tajikistan's Pamir Mountains. *Geoforum* **126**, 290-301 (2021).

3 Schewel, K. Understanding immobility: Moving beyond the mobility bias in migration studies. *International migration review* **54**, 328-355 (2020).

4 Upadhyay, H. Migration as good, bad and necessary: examining impacts of migration on staying Himalayan communities affected by climate change. *Humanities and Social Sciences Communications* **11** (2024).

Response to Reviewer 3

The paper address an important question: how does poverty interact with mobility in response to weather extremes. The paper indicates for Kyrgyzstan how wealth influences mobility, which is likely to be an important question in other jurisdictions. Wealth thresholds are divided into 3 based on the multidimensional poverty index MPI.

Some literature on mobility is missing or not referenced.

‘combined mobility’ should be described early in the paper – domestic mobility is not clearly explained (see below)

We thank the reviewer for the positive assessment and for highlighting the relevance of the wealth–mobility interaction beyond the Kyrgyzstan context.

In the revised manuscript, household well-being was operationalized using the Multidimensional Well-being Index (MWI), and resource thresholds were defined empirically by grouping households into terciles (low, middle, and high well-being). This categorization was intended as a transparent and data-driven way to capture heterogeneity in mobility constraints across the well-being distribution, rather than as a claim that mobility behavior was determined by three discrete poverty states. The tercile approach allowed us to illustrate non-linearities and substitution patterns in mobility outcomes while maintaining sufficient sample size within each group.

We also expanded the literature review to incorporate additional work on mobility, migration constraints, and immobility under climatic stress, ensuring closer engagement with relevant strands of the migration and climate–mobility literature in the introduction.

Finally, we clarified the definitions of mobility outcomes early in the paper. In particular, we explicitly defined domestic mobility and combined mobility in the Introduction and Methods sections. Combined mobility was clarified as referring to households reporting both domestic and international moves by different household members within the same observation period, rather than sequential or return migration by the same individual. These clarifications were added to improve transparency and interpretation of the results.

Strong points:

The paper introduces a new spatial model that layers household panel data with high-resolution climate records and spatial modelling to assess how multidimensional well-being (MWI), extreme weather events, and geography influence mobility in Kyrgyzstan.

We thank the reviewer for highlighting these strengths and for the positive assessment of the paper's methodological contribution and integration of household, climate, and spatial data. We appreciate this recognition.

For Kyrgyzstan, the paper highlights how weather related impacts in nearby districts inhibit migration for both the wealthy and poor households. This is an interesting point, as it shows that apart from just the resources to migrate/be mobile, structures for safe mobility can promote or inhibit mobility.

We thank the reviewer for this insightful interpretation. We agree that the spatial effects identified in the analysis highlight how structures and conditions for safe and feasible mobility, such as infrastructure, accessibility, and regional exposure to extreme weather, can promote or inhibit mobility beyond household resources alone. This interpretation is consistent with our framing of spatially correlated climate shocks as shaping local opportunity sets and mobility constraints.

Some issues to clarify earlier on:

Earlier on, where the authors tell us what the paper does, they should also tell us what it doesn't do. This comes later in Line 350 where the authors state that their model captures material and service-related dimensions but cannot fully reflect non-material factors such as social capital.

We thank the reviewer for this helpful suggestion. In the revised manuscript, we now clarify the scope and limitations of the analysis earlier in the paper, alongside the description of the paper's objectives and contributions.

Specifically, we explicitly state that the framework focuses on material and service-related dimensions of mobility capabilities, captured through household well-being, spatial accessibility, and exposure to weather shocks, and does not fully capture non-material factors such as social capital, governance quality, or migration policies. These limitations are therefore made clear at the outset, rather than only discussed later in the manuscript, to better manage expectations regarding the scope of the analysis.

While the paper introduces a new model, it overlooks a foundational literature on immobility and mobility, particularly work by De Haas, H. (2021) A theory of migration: the aspirations-capabilities framework. CMS 9, 8 (2021). https://doi.org/10.1186/s40878-020-00210-4

We thank the reviewer for highlighting the importance of the aspirations–capabilities literature. In the revised manuscript, we explicitly ground the analytical framework in this perspective and clarify its scope. As stated in the framework section, mobility outcomes are

conceptualized as reflecting the interaction between aspirations to move and capabilities to realize mobility, following de Haas (2021).

However, due to data limitations, migration aspirations are assumed to be present but are not directly observed or analyzed empirically. The empirical analysis therefore focuses deliberately on heterogeneity in mobility capabilities, shaped by household resources, health, access to services, and the ability to convert resources into effective action under climate stress. This distinction is now stated explicitly to avoid misinterpretation.

Accordingly, the resource-based mobility constraints framework is not intended as a full test of the aspirations–capabilities model, but as a spatially explicit empirical analysis of capability constraints in a climate-vulnerable, mountainous context.

The concept of 'combined migration' is difficult to understand and follow in the paper. This is particularly evident in a situation where the authors in one sentence say domestic mobility declined, while international mobility rose. In the next sentence, they argue that combined mobility became rare. So, the question becomes, "What is combined migration, and why does it matter?"

We thank the reviewer for this important clarification. In the revised manuscript, we explicitly define combined mobility as households reporting both domestic and international moves by different members within the same observation period (lines 431 - 434). It does not refer to sequential migration, return migration, or domestic relocation following international migration by the same individual.

With this definition, the patterns described in the Results are not contradictory. A decline in domestic mobility alongside an increase in international mobility reflects substitution across mobility types as higher fixed costs become affordable. Combined mobility remains rare because it requires sufficient household resources to support multiple mobility outcomes simultaneously, drawing on the same household resource pool. As constraints tighten, particularly under spatially correlated climate stress, households tend to prioritize a single mobility pathway rather than combining domestic and international moves.

Analytically, combined mobility matters because it highlights that mobility outcomes are jointly determined at the household level rather than independent individual decisions. Its rarity under climate stress provides additional evidence of binding resource constraints and supports the interpretation of mobility as a selective, resource-dependent response rather than a uniform or additive process. We clarify this interpretation in the revised text to improve transparency.

Include an overall statement about climate change in Kyrgyzstan

We thank the reviewer for this suggestion. In the revised manuscript, we added an explicit discussion of climate change and environmental stress in Kyrgyzstan in the Introduction. The revised text outlines key processes such as glacier retreat, increasing weather extremes, and their impacts on agricultural and pastoral livelihoods, and explains why Kyrgyzstan's high-altitude geography, reliance on climate-sensitive livelihoods, and dependence on labor migration make it a particularly relevant context for studying climate–mobility interactions.

The term domestic mobility is unclear. L174 Does this mean the whole household moves?

L109 what about movement within households, where some family members move first.

L184 Entire households do not always move abroad, some members move first, some cannot move because of age, some send members abroad first to diversity incomes, and often male household members move first or children are sent to relatives. Even with the extreme weather events mentioned in the paper, does the data indicate whether the entire household unit moves? L253 does mention this but only in terms of sending one member abroad while others relocate locally.

We thank the reviewer for these clarifications. In the revised manuscript, we explicitly clarify that domestic mobility does not imply that the entire household relocates. Rather, domestic and international mobility are defined at the household level and refer to cases in which one or more household members report an internal or cross-border move during the observation period. Combined mobility captures households in which different members engage in both domestic and international moves within the same period. These definitions are now stated explicitly and applied consistently throughout the paper.

Mobility indicators therefore include situations in which only some household members move, including cases where migration occurs in a staggered manner and others remain behind. This reflects common household-level mobility arrangements observed in Kyrgyzstan, without requiring the analysis to distinguish individual-level sequencing or roles.

Importantly, the KIHS does not allow us to distinguish entire-household relocation as a separate category, and such cases are likely rare. We therefore interpret mobility outcomes as reflecting household-level mobility strategies under shared resource constraints, rather than complete household relocation in response to extreme weather events. This interpretation is now clarified explicitly in the Methods and Results sections to avoid ambiguity.

Specific comments

Line 39 – apart from the costs of moving and adapting, what about access to relatives

We thank the reviewer for this clarification. We agree that access to relatives and kin networks is an important factor shaping mobility decisions, alongside the costs of moving and adapting. While the KIHS does not provide direct measures of access to relatives or kin-based support, these factors are discussed conceptually as part of the broader opportunity set that conditions households' ability to realize mobility.

In the revised manuscript, we clarify that such non-material factors, including access to relatives, social ties, and informal support networks, are not directly observed but may influence mobility indirectly through spatial spillovers, effective accessibility, and household well-being. We acknowledge these factors explicitly as important but beyond the scope of the available data.

Fig 1 explain what is meant by international threshold and international mobility. Whilst for Kyrgyzstan, it may mean neighbouring countries (Tajikistan, Uzbekistan, Kazakhstan…etc) but unclear does it mean beyond. More generally mobility across borders also depends on geography, borders (porous borders), and access. Although such information may not be available from the data set used.

We thank the reviewer for this clarification. In the revised manuscript, we clarify that international mobility refers broadly to cross-border migration, irrespective of destination. In the Kyrgyzstan context, this includes both migrations to neighboring countries and longer-distance international labor migration (most prominently to Russia). Figure 1 is intended as a conceptual illustration of relative mobility costs rather than a depiction of specific destinations.

The international mobility threshold therefore represents the higher resource requirements associated with crossing national borders—such as travel costs, documentation, information, and network access—relative to domestic mobility. We do not distinguish between different international destinations in the conceptual figure, as the purpose is to illustrate the ordering of mobility costs rather than country-specific migration regimes.

We agree that international mobility depends on geography, border regimes, and access, including the permeability of borders and institutional constraints. While such factors are not directly observed in the KIHS, they are implicitly reflected in the higher fixed costs of international mobility and in spatial accessibility measures included in the empirical analysis.

L63 –'enabling wealthier households to move abroad outward…" why just abroad here and what about intrahousehold movement. Rarely to entire households move abroad at once.

We thank the reviewer for this clarification. Throughout the manuscript, mobility is defined and analyzed at the household level, where domestic and international mobility refer to cases in which one or more household members relocate within Kyrgyzstan or across borders

during the observation period. We do not interpret international mobility as implying that entire households move abroad as a single unit.

While some expressions may refer generically to households "moving abroad," these are used as shorthand for household-level international mobility strategies involving individual members, consistent with the explicit definitions provided in the Methods section. We clarified these definitions early in the manuscript and apply them consistently to avoid ambiguity about intra-household and staged migration.

L70 Another factor is the geographical location of the country and national borders

We thank the reviewer for this clarification. In the manuscript, we discuss in detail how geography shapes mobility constraints in mountain regions, including rugged terrain, remoteness, sparse infrastructure, and long distances to markets and services, all of which raise the costs of relocation relative to household resources.

While we do not explicitly model national borders or border regimes, their relevance is implicitly captured through the higher fixed costs associated with international mobility and through the country context. Kyrgyzstan's landlocked position and mountainous geography condition access to international destinations and contribute to the elevated resource thresholds required for cross-border mobility. Given data limitations, border characteristics and migration policies cannot be analyzed directly and are therefore treated as part of the broader structural context rather than as separate explanatory variables. We clarify this interpretation to better situate the analysis.

L82 Geographic barriers AND geopolitical borders – there is an assumption that even if you want to move across a border and have the wealth, you can move.

We thank the reviewer for this clarification. This interpretation is emphasized in the conceptual framework section and the caption and discussion of Figure 1, where we clarify that international mobility entails substantially higher fixed costs than domestic mobility. These costs are explicitly interpreted as encompassing not only financial resources, but also institutional, geographic, and geopolitical constraints associated with crossing national borders.

In addition, in the framework discussion we state that international mobility becomes feasible only conditional on overcoming these cumulative constraints, rather than implying that sufficient wealth alone guarantees cross-border movement. While border regimes and migration policies cannot be modeled explicitly with the available data, they are treated as part of the structural constraints embedded in the international mobility threshold.

L191-193 makes an important point about regional shocks flattening differences in mobility across districts, which would indicate that domestic mobility is quite local or regional first?

We thank the reviewer for this insightful interpretation. The finding that regional shocks flatten differences in mobility across districts is consistent with the idea that domestic mobility is often local or regional in nature, particularly in the short to medium term. When weather shocks affect multiple neighboring districts simultaneously, they reduce spatial variation in local opportunities, which can dampen domestic mobility responses across the affected region.

However, the data do not allow us to observe the precise distance or destination of domestic moves. We therefore interpret this result more cautiously as evidence that region-wide shocks constrain local opportunity sets, rather than as definitive proof that domestic mobility is limited to very short distances. This interpretation aligns with our framing of spatially correlated shocks as shaping household mobility strategies through shared constraints rather than through isolated district-level effects.

L219 -221 first sentence -… "it does not capture the additional effects of compound extreme weather events.

We thank the reviewer for this comment. In the revised manuscript, we clarified that the empirical specification did not model compound extreme weather events explicitly (Methods section, lines 219–221). At the same time, we addressed concerns regarding potential confounding from concurrent weather extremes through diagnostic analyses.

Prior to estimation, we explicitly assessed overlap and statistical dependence among weather indicators. Although multiple extremes occasionally occurred in the same year, higher-order concurrence was rare. Pairwise correlations among climate variables were modest ($|\rho| \leq 0.20$), and variance inflation factors remained close to unity ($VIF \leq 1.09$), indicating no multicollinearity concerns. These diagnostics confirmed that the estimated associations were not driven by overlapping extreme events. We reported and discussed these results in the robustness section and Supplementary Tables S8–S9 (lines 599–606).

We therefore interpreted the results as reflecting the marginal associations of distinct climate extremes rather than compounded or interacting hazard effects and noted the explicit modeling of compound events as an important direction for future research.

L266 – 267 add at the end – of 'do not produce a uniform mobility outcome.. of household units? Or of households

We thank the reviewer for this clarification. The statement referred to households, which were the unit of analysis throughout the paper. In the revised manuscript, we clarified the

wording to state explicitly that extreme weather events did not produce a uniform mobility outcome across households, rather than implying household units or individuals ambiguously.

Discussion and Concluding remarks

Might be good to mention planned relocation options?

They exist in other countries, do they exist in Kyrgyzstan? Is it likely that the government will engage in planned relocation options in the future? if so why or why not?

We thank the reviewer for this valuable suggestion. In the revised manuscript, we expanded the Discussion to situate our findings in relation to planned relocation as one possible, but context-dependent, adaptation pathway.

We clarified that, while planned relocation has been implemented or discussed as a policy option in several climate-vulnerable settings internationally, it is not currently a prominent or institutionalized adaptation strategy in Kyrgyzstan. Given the country's strong reliance on labor mobility as a livelihood strategy, limited fiscal capacity, and the social and cultural importance of place-based livelihoods—particularly in high-altitude pastoral regions—large-scale planned relocation is unlikely to represent a near-term or primary policy response.

Consistent with this context, we emphasized that the policy implications of our findings point more strongly toward supporting both mobility and safe immobility in situ. In particular, spatially widespread extreme weather events can constrain mobility options themselves, underscoring the importance of scalable in-country adaptation measures such as social assistance, healthcare provision, and emergency services in remote and high-elevation areas. We framed planned relocation as a potential long-term or exceptional option under severe and persistent risk, rather than a general substitute for mobility- or development-based adaptation.

We therefore positioned planned relocation as part of a broader adaptation discussion, while emphasizing that, in the Kyrgyz context, strengthening human development, social protection, and coordinated regional adaptation remains the most plausible and equitable policy pathway under increasing climate risk.

Response to Reviewer 1

The authors have provided a response to the previous review and have implemented a number of revisions. The discussion of the spatial specification, the treatment of climate variables, and the description of the survey design are improved. The manuscript now offers clearer explanations of the empirical strategy, additional robustness checks, and improved explanation regarding the rotating panel structure of the KIHS dataset and the spatial modelling approach. These revisions improves the clarity and interpretability of the analysis.

We thank the reviewer for the positive and constructive assessment of the revised manuscript. We appreciate the recognition of the improvements in the empirical strategy, treatment of climate variables, and overall clarity of the analysis.

However, a few aspects could still benefit from further clarification:

(i) The authors note that the empirical results should be interpreted as conditional associations rather than causal estimates. This clarification is welcome. Nonetheless, the manuscript could briefly acknowledge that lagging the well-being index may not fully eliminate potential feedback mechanisms between migration and household welfare, for example through remittances or labor reallocation.

We thank the reviewer for this helpful clarification. We agree that lagging the multidimensional well-being index (MWI) mitigates simultaneity concerns but does not fully eliminate potential feedback mechanisms from migration to household welfare (e.g., via remittances or labor reallocation).

We have revised the manuscript to acknowledge this limitation more explicitly in the *Econometric strategy* (lines, 526-528) and *Robustness and sensitivity* (lines, 555-558) sections. Accordingly, we continue to interpret the results as conditional associations rather than causal effects.

(ii) The explanation of the spatial modelling framework is much clearer in the revised version, and the additional diagnostics are helpful. At the same time, it may be useful to acknowledge that some of the spatial correlation observed in the results may reflect shared regional exposure to climate conditions rather than direct spillover mechanisms across districts.

We thank the reviewer for this insightful comment. We agree that spatial correlation in mobility outcomes may reflect not only spillover mechanisms across districts, but also shared regional exposure to correlated climatic conditions.

We have revised the manuscript to clarify this interpretation in two places. First, in the *Econometric strategy* section (lines, 511-513), we now note that spatially lagged climate variables capture spatially structured exposure across neighboring districts arising from both common regional climate patterns and indirect spillover processes. Second, in the *Robustness and sensitivity* section (lines, 612-616), we clarify that the observed spatial correlation should not be interpreted solely as evidence of direct spillovers, as neighboring districts may experience correlated climatic conditions.

Response to Reviewer 2

The authors have responded carefully and constructively to the reviewer comments, and the revised manuscript is substantially improved as a result. The revised manuscript is substantially improved. The revisions demonstrate a thoughtful engagement with the critiques, and many of the earlier concerns have been addressed through clearer conceptual framing, more cautious interpretation of results, and improved transparency in the methodological description.

I found the authors have convincingly moderated the language around causal inference. The revised manuscript now consistently frames the results as conditional associations rather than causal effects, which is appropriate given the observational nature of the data and the spatial econometric approach used. The explicit discussion of endogeneity concerns and methodological limitations enhances the analytical credibility of the paper.

The authors have made a meaningful effort to broaden the conceptual framing beyond a purely wealth-centered interpretation. By situating the analysis within the aspirations–capabilities framework and acknowledging the role of social networks, governance structures, migration regimes, and cultural attachments, the manuscript now better reflects the complexity of migration decision-making. Although these factors cannot be directly modeled with the available data, the authors appropriately recognize their importance and clearly delimit the empirical scope of the study.

The manuscript now provides much clearer justification for methodological choices, including the construction of the Multidimensional Well-being Index (MWI), the treatment of climate indicators, and the specification of spatial weights. These revisions significantly improve the transparency and reproducibility of the study.

The discussion of mountain-region comparability has been expanded and now situates the Kyrgyzstan case more convincingly within the broader literature on mobility. This helps clarify which mechanisms may be generalizable (e.g., resource thresholds, spatial exposure, and mobility costs) and which remain context-specific.

Authors also carefully revised the policy implications section, which is much stronger and more contextually grounded. The authors now link their findings directly to Kyrgyzstan’s institutional context, including labor migration arrangements with Russia and Kazakhstan, spatially targeted social protection in mountain districts, and the need for coordinated adaptation planning across neighboring administrative units. These additions enhance the study's practical relevance.

In addition, the manuscript now more clearly addresses voluntary immobility and place attachment, acknowledging that immobility in mountain communities can also stem from cultural ties and identity-based connections to land. The clarification that the empirical framework focuses on capabilities rather than aspirations is an important conceptual improvement.

Overall, the revisions significantly strengthen the manuscript's clarity, conceptual balance, and methodological transparency, while preserving its original empirical contribution. The study remains an ambitious and valuable contribution to the literature on climate-related mobility, particularly in mountainous regions where empirical evidence is still limited.

We sincerely thank the reviewer for the very positive and thoughtful assessment of the revised manuscript. We greatly appreciate the recognition of the improvements in conceptual framing, methodological transparency, and the more cautious interpretation of results.

We are particularly encouraged that the revisions regarding the aspirations–capabilities framework, treatment of endogeneity, and the contextualization of findings and policy implications have strengthened the manuscript. In this final revision, we have made a few additional clarifications in response to the remaining reviewer comments, while preserving the overall structure and contributions of the study.

Response to Reviewer 3

The authors have responded to most of my previous comments and the manuscript has improved in several respects. In particular, the discussion of different types of migration is clearer, and the paper now provides a more explicit explanation of whether migration occurs at the individual or household level. The expanded discussion on destinations and labour markets is also a welcome addition, as it helps situate migration decisions within broader economic contexts.

We thank the reviewer for the constructive feedback and for recognizing the improvements in the revised manuscript, particularly regarding the clarification of migration types and the discussion of destinations and labor market context.

However, I remain unclear about how the concept of wellbeing is defined and operationalised in the paper. At present, the analysis appears to treat wellbeing primarily as a function of wealth or economic outcomes. If this is the intended conceptualisation, the authors should state this explicitly and justify the choice with reference to the relevant literature. Alternatively, if wellbeing is intended not to encompass broader dimensions apart from livelihoods and wealth (such as social, psychological… factors.) this should be clarified and reflected more clearly in the analysis and discussion.

We appreciate this important comment. We agree that the concept of well-being required clearer definition and positioning. In the revised manuscript, we now explicitly clarify that the Multidimensional Well-being Index (MWI) captures a bounded set of capability-relevant living conditions—nutrition, education, health, and living standards—rather than the full spectrum of well-being, such as social or psychological dimensions.

We emphasize that the index reflects material and service-based conditions that shape households' capabilities for mobility, rather than subjective or relational aspects of welfare. This clarification has been added in the *Key variables* section (lines, 454-459) and reinforced in the *Discussion* (lines, 316-317).

I am not in a position to assess the econometric strategy employed in the paper and will defer to other reviewers who are better placed to evaluate the technical aspects of the methodology.

Figure 2 would benefit from additional explanation in both the main text and the figure caption. It is not entirely clear whether Figure 2 is intended to represent a hypothesis, a conceptual framework, and seems to be an illustration of the relationships discussed in the paper. Clarifying the purpose of the figure would help readers better understand how it should

be interpreted. While I can follow the description provided in the text, the meaning of the purple arrow, labelled "substitution under shared resource constraint," remains unclear in the visual representation. The authors should clarify how this relationship is conceptualised and how it should be interpreted within the framework presented in the figure. In addition, the origin and purpose of the dotted lines are not immediately evident are they based on data? It is important that readers can easily understand the relationships being illustrated.

We thank the reviewer for highlighting the need to clarify the role and interpretation of Figure 2. We have revised both the main text and the figure caption to make explicit that Figure 2 is a conceptual illustration of the resource-threshold framework, rather than an empirical result or formal hypothesis test.

In addition, we now clarify that the purple arrow represents substitution across mobility outcomes under a shared household resource constraint, reflecting the reallocation of limited resources from domestic to international mobility as capabilities increase. We also explain that the dashed curves and vertical lines illustrate stylized shifts in mobility thresholds and outcomes under extreme weather conditions—through both push effects and constraint mechanisms—and are conceptual rather than based on observed data.

These clarifications have been incorporated in the paragraph introducing Figure 2 (lines, 88-89 and 95-97) and in the revised figure caption.

Response to Reviewer 3

The revised text tackles the reviewers comments and is much improved.

We thank Reviewer #3 for the positive assessment of the revised manuscript and for the helpful additional suggestions.

I feel the order of the abstract is not quite there. Also the abstract the sentence that starts with "we find that extreme weather events, both locally and in neighboring districts is most strongly associated with international mobility and immobility"... " does not make sense.

We thank the reviewer for this helpful observation. We revised the abstract to improve its overall flow and ordering, particularly by clarifying the presentation of the main findings. We also rephrased the sentence referring to local and neighboring weather extremes and their association with different mobility outcomes, which was previously unclear. The revised abstract now more clearly distinguishes the relationships between weather exposure and the four mobility outcomes examined in the study.

Figure 2 is clearer

We appreciate the reviewer's positive feedback regarding the improved clarity of Figure 2.

Table 1 require more explanation - either with better header, more explanations and a description of that the table is telling us overall.

We thank the reviewer for this helpful suggestion. To improve the interpretability of Table 1, we revised the accompanying text to more clearly highlight the main descriptive patterns across elevation groups, mobility outcomes, household well-being, and exposure to weather extremes. Specifically, we integrated a clearer discussion of the spatial concentration of immobility and low household well-being in high-altitude areas, the relative prominence of international mobility in mid-elevation districts, and the overlap between climatic exposure and socioeconomic vulnerability. We also revised the structure of the descriptive results section to improve the overall narrative flow and interpretation of the table.
